# Data science assisted investigation of catalytically active copper hydrate in zeolites for direct oxidation of methane to methanol using H_2_O_2_

**DOI:** 10.1038/s41598-021-81403-4

**Published:** 2021-01-22

**Authors:** Junya Ohyama, Airi Hirayama, Nahoko Kondou, Hiroshi Yoshida, Masato Machida, Shun Nishimura, Kenji Hirai, Itsuki Miyazato, Keisuke Takahashi

**Affiliations:** 1 Faculty of Advanced Science and Technology, Kumamoto University, 2-39-1 Kurokami, Chuo-ku, Kumamoto, 860-8555 Japan; 2 Department of Applied Chemistry and Biochemistry, Graduate School of Science and Technology, Kumamoto University, 2-39-1 Kurokami, Chuo-ku, Kumamoto, 860-8555 Japan; 3 Graduate School of Advanced Science and Technology, Japan Advanced Institute of Science and Technology (JAIST), 1-1 Asahidai, Nomi, 923-1292 Japan; 4 Research Institute for Electronic Science, Hokkaido University, N20W10, Kita-Ward, Sapporo, 001-0020 Japan; 5 Department of Chemistry, Hokkaido University, N-15 W-8, Sapporo, 060-0815 Japan

**Keywords:** Cheminformatics, Physical chemistry, Catalysis, Heterogeneous catalysis

## Abstract

Dozens of Cu zeolites with MOR, FAU, BEA, FER, CHA and MFI frameworks are tested for direct oxidation of CH_4_ to CH_3_OH using H_2_O_2_ as oxidant. To investigate the active structures of the Cu zeolites, 15 structural variables, which describe the features of the zeolite framework and reflect the composition, the surface area and the local structure of the Cu zeolite active site, are collected from the Database of Zeolite Structures of the International Zeolite Association (IZA). Also analytical studies based on inductively coupled plasma-optical emission spectrometry (ICP-OES), X-ray fluorescence (XRF), N_2_ adsorption specific surface area measurement and X-ray absorption fine structure (XAFS) spectral measurement are performed. The relationships between catalytic activity and the structural variables are subsequently revealed by data science techniques, specifically, classification using unsupervised and supervised machine learning and data visualization using pairwise correlation. Based on the unveiled relationships and a detailed analysis of the XAFS spectra, the local structures of the Cu zeolites with high activity are proposed.

## Introduction

Active site structures in catalyst materials including heterogeneous catalysts, complexes, and enzymes are of great interest in chemistry and chemical industry, because new advanced catalyst materials can be designed based on the active structures^1–5^. In order to reveal the active site structures, it is necessary to investigate structure–activity relationships carefully based on various structural data of catalysts describing them accurately and thoroughly. Here, the data science provides powerful approaches to understand the complex structure–activity relationships and to identify the key structural parameters^6–9^.

The active site structures of methane monooxygenase (MMO) have been studied intensively because they offer direct oxidation of CH_4_ to CH_3_OH even at ambient temperature and pressure. MMO has two well-known forms, particulate MMO (pMMO) and soluble MMO (sMMO) which have active sites of Cu and Fe, respectively^10^. The structure of the Cu active sites in pMMO is still controversial as mono-nuclear or bi-nuclear Cu species have been proposed as highly active sites for CH_4_ oxidation^1,11,12^. Meanwhile, pMMO-inspired Cu active sites have been developed not only in complex catalysts but also in heterogeneous solid catalysts, more specifically, in zeolite cages^5,11,13,14^.

Cu zeolites are considered to be one of the most promising candidate catalyst materials for direct oxidation of CH_4_ to CH_3_OH in industrial processes^15^. Previous studies have demonstrated CH_3_OH production using various oxidants including H_2_O_2_, NO_x_, O_2_ and H_2_O^16–22^. When Cu zeolites are applied to CH_4_ oxidation using H_2_O_2_ in batch type reactors, CH_3_OH and CH_3_OOH are selectively produced while overoxidation to HCOOH and CO_2_ are suppressed^16,23,24^. The problem of this process is the high cost of H_2_O_2_ which is more expensive than CH_3_OH, although the reaction using H_2_O_2_ give higher productivity and selectivity of CH_3_OH than the reactions using the other oxidants. In the case of CH_4_ oxidation using O_2_, Cu zeolites hardly act as catalysts, but activated Cu zeolites, prepared by heat treatment under O_2_ offer methoxy species on active sites by stoichiometric reaction with CH_4_. Then, the methoxy species are treated by water vapor to extract CH_3_OH. Thus, the chemical looping process involving Cu zeolite activation, CH_4_ oxidation and CH_3_OH extraction has been proposed for direct conversion of CH_4_ to CH_3_OH^25,26^. Recent analysis from an economic standpoint has suggested that the problem of the chemical looping process lies in the production efficiency of CH_3_OH and the durability of Cu zeolites^15^. On the other hand, when NO_x_ is used as an oxidant, CH_3_OH is formed over Cu zeolites continuously in gas flow reactors; however, the CH_3_OH selectivity is much lower than those of the former two processes^18,27^. Thus, methods for direct oxidation of CH_4_ to CH_3_OH need to be improved further for practical application.

The local structure around the Cu sites in zeolites may be the key to catalytic activity for CH_4_ oxidation, because the local structure strongly influences the adsorption of reactants, intermediates and products, and consequently reaction results such as reactant conversion and product selectivity are considerably affected^28,29^. In addition, zeolite-framework-derived diffusion and adsorption of molecules can influence the reaction results^30,31^. Thus, it is necessary to gain an improved understanding of catalytic reactions by investigating structure–activity relationships based on various structural data which describe each catalyst accurately. Furthermore, if the key structural descriptors and their effects are revealed, new catalysts can be developed based on fundamental structural design considerations. Here, the data analysis techniques developed in data science such as machine learning and data visualization, are considered useful for revealing the key descriptors in “hidden” relationships in complex multidimensional data^6,7^. Recently, such data analysis techniques have been applied in the field of catalysis chemistry^7,32–41^. Meanwhile, the construction and publication of databases related to catalyst materials are also progressing. As for zeolites, the Structure Commission of the International Zeolite Association (IZA-SC) has provided and upgraded structural data for all zeolite framework types since 1996^42^. Consequently, data for various materials can be explored as catalyst descriptors.

Measured structural data often become more important for describing actually used catalysts than the common data obtained from the published databases. In the case of Cu zeolites, UV–Vis spectra, X-ray diffraction and X-ray absorption fine structure (XAFS) spectra are analyzed as they reflect local structures of Cu active sites^14,29,43^. XAFS spectra provide sensitive and accurate information on valence, symmetry and coordination structure of Cu active sites. Thus, Cu K-edge XAFS has been used to reveal the structure of Cu active sites for CH_4_ oxidation as well as that for NO_x_ purification^14,29,43^. It should also be noted that advances in synchrotron radiation and optical techniques in recent decades have permitted collection of XAFS spectra in a relatively short period of time^44^. Therefore, the actual structural data of active sites can be effectively obtained by XAFS measurement.

Here, dozens of Cu zeolites are prepared and CH_4_ oxidation is performed in a batch type reactor using H_2_O_2_ as an oxidant. Information on the zeolite framework structure is collected from the database. In addition, Cu K-edge XAFS spectra and specific surface areas are obtained for the prepared catalysts. Based on the dataset consisting of reaction and structural data, the active site structures in the Cu zeolite catalysts for CH_4_ oxidation are investigated with the aid of data science techniques.

## Methods

### Catalyst preparation

CHA type zeolites and JRC-Z90 are obtained from JGC Catalysts and Chemicals Ltd. and the Catalysis Society of Japan, respectively. The other zeolites are provided by Tosoh Corporation. Copper-exchanged zeolites are prepared by adding 1–2 g of zeolite powder to aqueous solutions of Cu(CH_3_COO)_2_·H_2_O. After stirring at 80 °C for 3 h, the suspensions are filtered, washed with water, and dried at 110 °C overnight. The samples are calcined at 700 °C for 1 h. The zeolites are designated by M(X)-TYP-Y where M is the exchanged metal species, (X) is the loading amount of M, TYP is the 3-letter code which indicates the type of framework for the zeolite and Y is the Si/Al_2_ ratio. Fe-MFI(37) and Mn-MFI(39) are also prepared by the same ion exchange method using Fe(CH_3_COO)_2_, Mn(CH_3_COO)_2_·4H_2_O and H-MFI(37) or H-MFI(39) at (M^n+^/mol)/(Al/mol) = 1; Co-MFI(39) is prepared using Co(CH_3_COO)_2_·4H_2_O and NH_4_-MFI(39) at (M^n+^/mol)/(Al/mol) = 1; and Ni-MFI(37) is prepared using Ni(CH_3_COO)_2_·4H_2_O and H-MFI(37) at (M^n+^/mol)/(Al/mol) = 0.5. Ag-MFI(37) is prepared using AgNO_3_ and 1 g of H-MFI(39) by a similar ion exchange method performed at 45 °C for 2 h, followed by calcination at 550 °C for 4 h. Rh-MFI(22.5) is prepared by impregnation of NH_4_-MFI(22.5) in Rh(NO_3_)_3_ solution to be 0.5wt% Rh loading, followed by calcination at 550 °C for 3 h.

### Characterization

The specific surface area of samples is determined by N_2_ adsorption using the Brunauer–Emmett–Teller (BET) equation on BELSORP-mini (MicrotracBEL Corp.).

The Cu loadings of Cu zeolites are determined using inductively coupled plasma-optical emission spectroscopy (ICP-OES, Thermo iCAP7400) and X-ray fluorescence (XRF, Rigaku EDXL 300). The aqueous solutions for ICP analysis are prepared by a fusion method as reported elsewhere^45^. The mixture of 20 mg of Cu zeolite and 0.5 g of sodium peroxide is heated in a Zr crucible at 500 °C. The resulting molten samples are dissolved by adding 20 mL of 2 M HCl. The Cu loading is calculated from the Cu/Al evaluated by the ICP-OES measurement and the Si/Al specified on the manufacturer’s catalogue. The Cu loadings are also evaluated from XRF analysis of the Cu zeolite powders under He flow or under evacuation. Figure S1 shows the relationship between the relative XRF intensities for Cu/(Cu + Al + Si) and the Cu loadings determined by ICP-OES, where the data for 26 samples are plotted. Using the linear relationship and the relative XRF intensities, the Cu loadings for 35 Cu zeolite samples are determined.

Cu K-edge XAFS measurements are performed on the BL14B2 at SPring-8 for two Cu-CHA samples and on BL11 and BL15 at the SAGA Light Source for the remaining Cu zeolite samples. The XAFS spectra are taken using a conventional transmission method with a Si(111) double crystal monochromator and ion chambers. Data are analyzed using the Athena software including in the Demeter package^46^. The energies of all spectra are calibrated by using the spectra of Cu foil. The Fourier transform of the extended XAFS (EXAFS) is performed for *k* = 3–10. An EXAFS simulation using FEFF6 is conducted in the Arthemis software, where two kinds of Cu–O scatterings are calculated with a Debye–Waller factor of 0.003 and with summing up to construct model local structures for Cu^2+^(H_2_O)_x_ (x = 4–6)^46,47^.

### Data analysis using data science techniques

Scikit–learn (version 0.17) and pandas are implemented for supervised and unsupervised machine learning as well as for calculation of pairwise correlation of the variables representing the structure and reactivity of the Cu zeolites^48^. A Gaussian mixture model within unsupervised machine learning is used for classifying the data where the covariance type is set to full. Random forest classification, supervised machine learning, is used to evaluate the importance of descriptors^49^. The number of trees in the random forest is set to 100 where the random state with the highest score is chosen. Cross validation is used to evaluate the accuracy of each machine learning algorithm where the data are split into test data (20% of the data) and trained data (80% of the data). The average score of ten random tests is evaluated. The pairwise correlations are calculated to evaluate the correlations between the variables.

### CH_4_–H_2_O_2_ batch reaction

Prior to the CH_4_–H_2_O_2_ reaction, the catalysts are calcined at 700 °C for 1 h and then cooled to room temperature under an ambient atmosphere. The CH_4_–H_2_O_2_ reaction is performed in an autoclave (20 mL, TVS-1 type, Taiatsu Techno Co.) with a glass inner tube. 10 mg of catalyst powder is added to 3.0 mL of water in the glass inner tube, where a small polyethylene cup containing 155 μL of 30% H_2_O_2_ aq is dispensed^50^. After the glass tube is positioned in the stainless steel autoclave, the autoclave is filled with 5.0 MPa of N_2_ for a leak check, then replaced with 4.0 MPa of CH_4_ three times, and then charged with 3.5 MPa of CH_4_. After the autoclave is heated to 60 °C, the reaction is started by adding H_2_O_2_ aq to the catalyst suspension by shaking the autoclave to turn over the cap containing the H_2_O_2_. The reaction solution is stirred at 700 rpm for 1 h. The suspension after the reaction is filtered and 700 μL of the filtrate is mixed with 200 μL of D_2_O for product quantification using ^1^H-NMR (JEOL JNM-ECZ400R). The mixed liquid is transferred to an NMR glass tube (3 mm diameter) with an inner tube containing about 0.03% tetramethylsilane (TMS) solution in CDCl_3_. The ^1^H NMR spectra are obtained using a water suppression pulse program. The reaction products, i.e., CH_3_OH, CH_3_OOH and formic acid, are quantified from the peak areas at δ = 3.36, 3.87 and 8.26 ppm, respectively, by the external standard method using maleic acid as standard. The gas phase in the autoclave is analysed by a gas chromatograph (GC-2014, Shimadzu) equipped with a thermal conductivity detector.

## Results and discussion

Thirty-five Cu zeolites and twenty H zeolites are tested for the CH_4_–H_2_O_2_ reaction in the batch reaction system and the results are presented in Fig. S2a,b and Table S1. CH_3_OH, CH_3_OOH, or HCOOH are observed as the products, and the main product is varied with the catalysts (Figs. S2a, S3, Table S2). The Cu zeolites offer CH_3_OH and CH_3_OOH as the oxygenated products (Figs. S2a, S3c,d). When H-MFI zeolites are used, the overoxidized product, HCOOH, is formed in addition to CH_3_OH and CH_3_OOH (Figs. S2b, S3a). Thus, the Cu^2+^ in Cu-MFI suppresses overoxidation of CH_4_, which is consistent with previous studies^16,23,24^. The catalytic activity of H-MFI is attributed to Fe contamination which has activity for non-selective oxidation of CH_4_ via the Fenton reaction (Table S2)^24^. Interestingly, the other H-zeolites of MOR, FER, FAU and CHA show much less oxidized products than H-MFI, and do not produce HCOOH (Figs. S2b, S3b). This might be due to the difference in the structure of the Fe species in the H-zeolites (Fig. S4). In the viewpoint of H_2_O_2_ utilization, the different Fe and Cu species do not cause significant change in the H_2_O_2_ utilization based on the H_2_O_2_ concentration measurement after the CH_4_–H_2_O_2_ reaction using several zeolites (Fig. S5). The CH_4_-H_2_O_2_ reaction is also performed using other metal exchanged zeolites, i.e., Fe, Co, Ni, Rh and Ag-MFI. As presented in Table S3, the M-MFI other than Cu-MFI produce HCOOH. According to the literature, CH_3_OH is formed by the decomposition of CH_3_OOH, while HCOOH is formed by overoxidation of CH_3_OH or non-selective oxidation^24^. Therefore, Cu species are considered to be effective for selective oxidation of CH_4_ to CH_3_OH and CH_3_OOH. The result is in good agreement with previous studies^16,23,24^.

**Figure 1:**
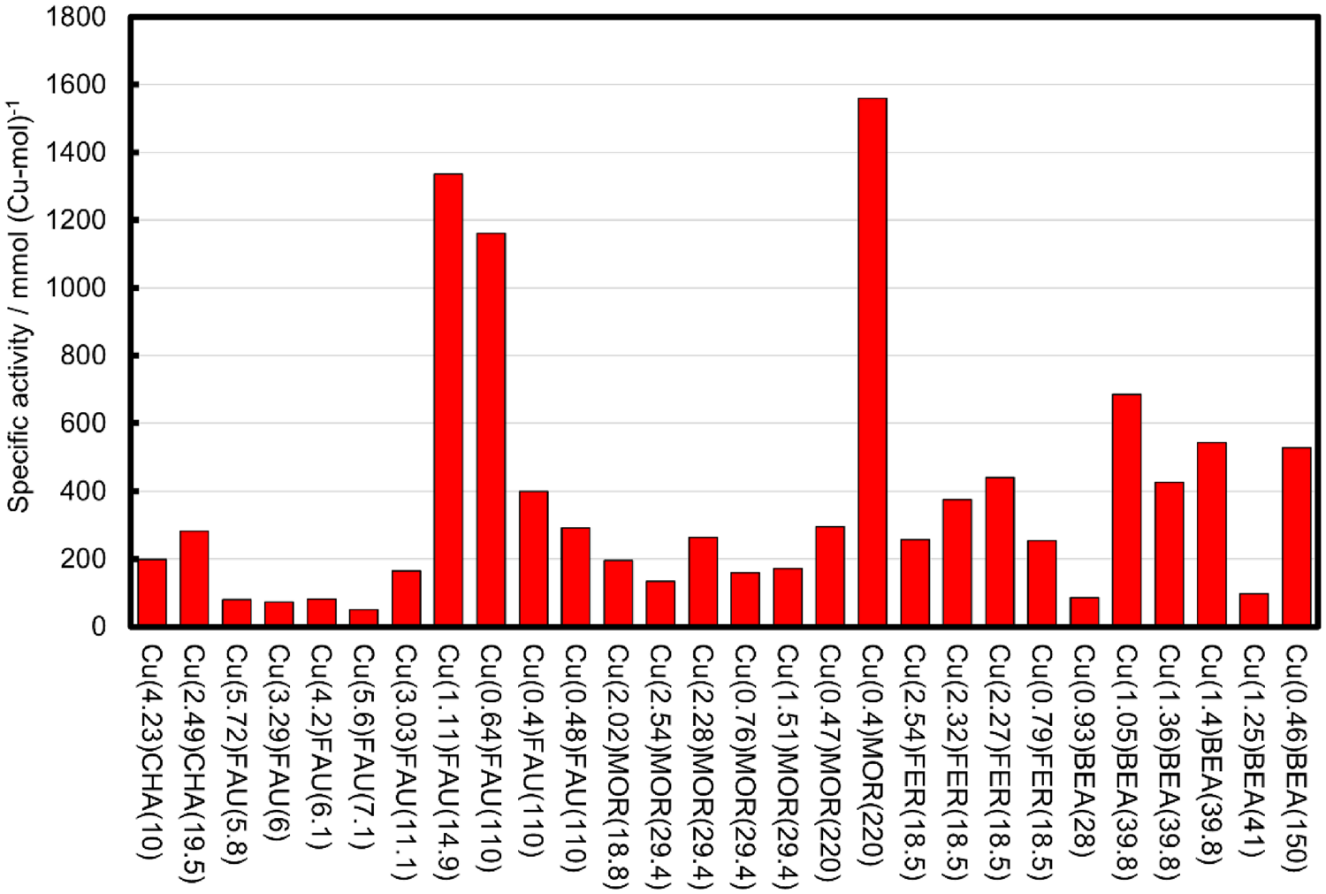
Specific activity of twenty-eight Cu zeolites for the CH_4_–H_2_O_2_ reaction. Reaction conditions: CH_4_ 3.5 MPa, 30 wt% H_2_O_2_ 155 μL, H_2_O 3 mL, catalyst 10 mg, 60 °C, 1 h. The specific activity is determined by dividing the product increments shown in Fig. S2c with the amount of Cu listed in Table S1.

To investigate the catalytic performance of Cu species in the zeolites, the increments of the products due to Cu exchange are evaluated from the differences in total yields of all products before and after Cu exchange. The product increments for all Cu zeolites are shown in Fig. S2c, where all Cu-MFI show negative values. It is reasonable to consider that Cu in the MFI zeolites traps oxygen radical species as a result of Fe contamination in the MFI zeolites. A more important fact is that the other Cu-zeolites show positive values (Fig. S2c), which are attributable to catalysis by Cu species. On the other hand, the catalysis of Cu species in MFI cannot be evaluated because of the too strong influence of Fe species in MFI on the catalytic performance. Therefore, active structures of Cu zeolites can be investigated due to the catalytic activity of Cu-zeolites other than Cu-MFI. It should be also noted that neither CO_2_ nor CO (< 6 ppm) is detected in the gas phase after the CH_4_–H_2_O_2_ reaction using several catalysts including H-MOR(18.8), H-MOR(29.4), and Cu(2.02)-MOR(18.8) by a gas chromatograph with a thermal conductivity detector. Thus, the catalyst activity is evaluated from the total products of CH_3_OH and CH_3_OOH. Figure 1 shows the specific activity determined by dividing the product increments by the amount of Cu in the Cu zeolites. The catalyst activity varies with the Cu zeolites, suggesting that the catalyst activity varies depending on the Cu zeolite structure.

The catalyst structural data are collected in order to explore the highly active structures. Table 1 lists the catalyst structural data collected in this study. The structural data due to the zeolite framework type are taken from the Database of Zeolite Structures of the IZA^42^. More specifically, the following eight variables are collected: framework density; topological density (TD10); channel dimensionality (CD); maximum diameter of a sphere that can be included; those that can diffuse along three unit vectors (Da, Db, Dc); and accessible volumes. The surface area, which also belongs to the zeolite structural data, is evaluated by N_2_ adsorption measurements. Further, the zeolite compositional data includes the Si/Al_2_ ratio of zeolite, the Cu loadings and the ion exchange rates (Cu/Al_2_ ratio, denoted as IE) which are determined by the ICP/XRF measurements.

**Table 1:** Structural data collected as catalyst descriptors.

Feature	Variable (abbreviation)	Source or analysis method
Zeolite framework	Framework density (FD); topological density (TD10); maximum diameter of a sphere that can be included (DI); those can diffuse along three unit vectors (Da, Db, Dc); accessible volumes (AV), channel dimensionality (CD)	Database of zeolite structures of the IZA
Composition	Si/Al_2_ ratio of zeolite (Si/Al_2_); Cu loading (Cu wt); ion exchange rate (IE)	ICP, XRF
Surface area	Surface area (SA)	N_2_ adsorption
Local structure of Cu	X-ray absorption edge energy at 0.5 of normalized absorption (E at abs 0.5); FT-EXAFS peak intensity at ca. 1.5 Å (Int at 1.5 Å); that at ca. 2.1 Å (Int at 2.1 Å)	XAFS

**Figure 2:**
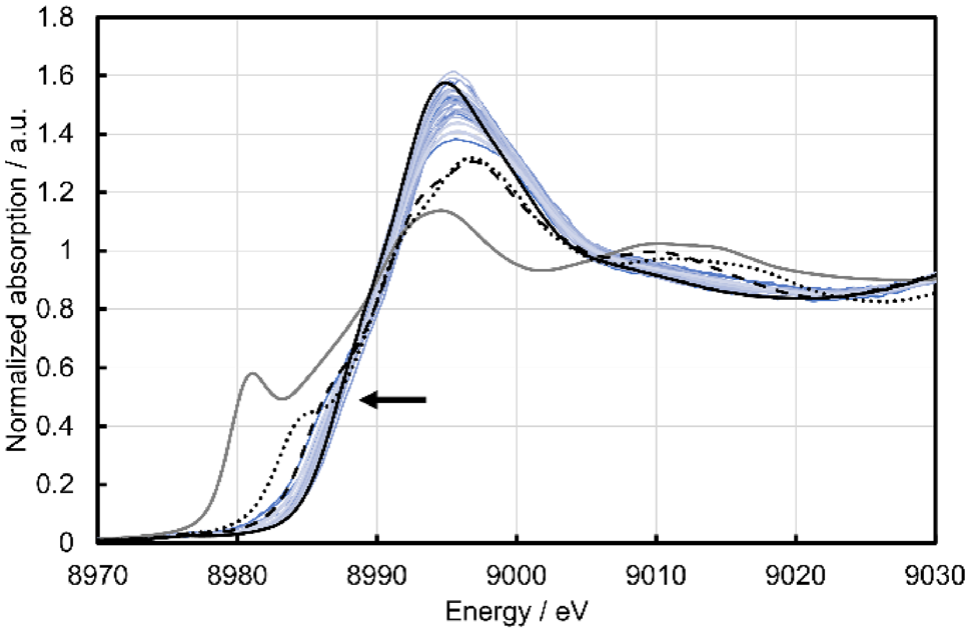
Cu K-edge XANES spectra of the twenty-eight Cu zeolites presented in Fig. 1 (blue), together with reference spectra for Cu_2_O (gray solid), Cu(OH)_2_ (black dashed), CuO (black dotted) and Cu(NO_3_)_2_ aq (black solid). The arrow indicates the adsorption edge at 0.5 of the normalized absorption, where the edge energies are collected as a structural variable of the Cu zeolites.

**Figure 3:**
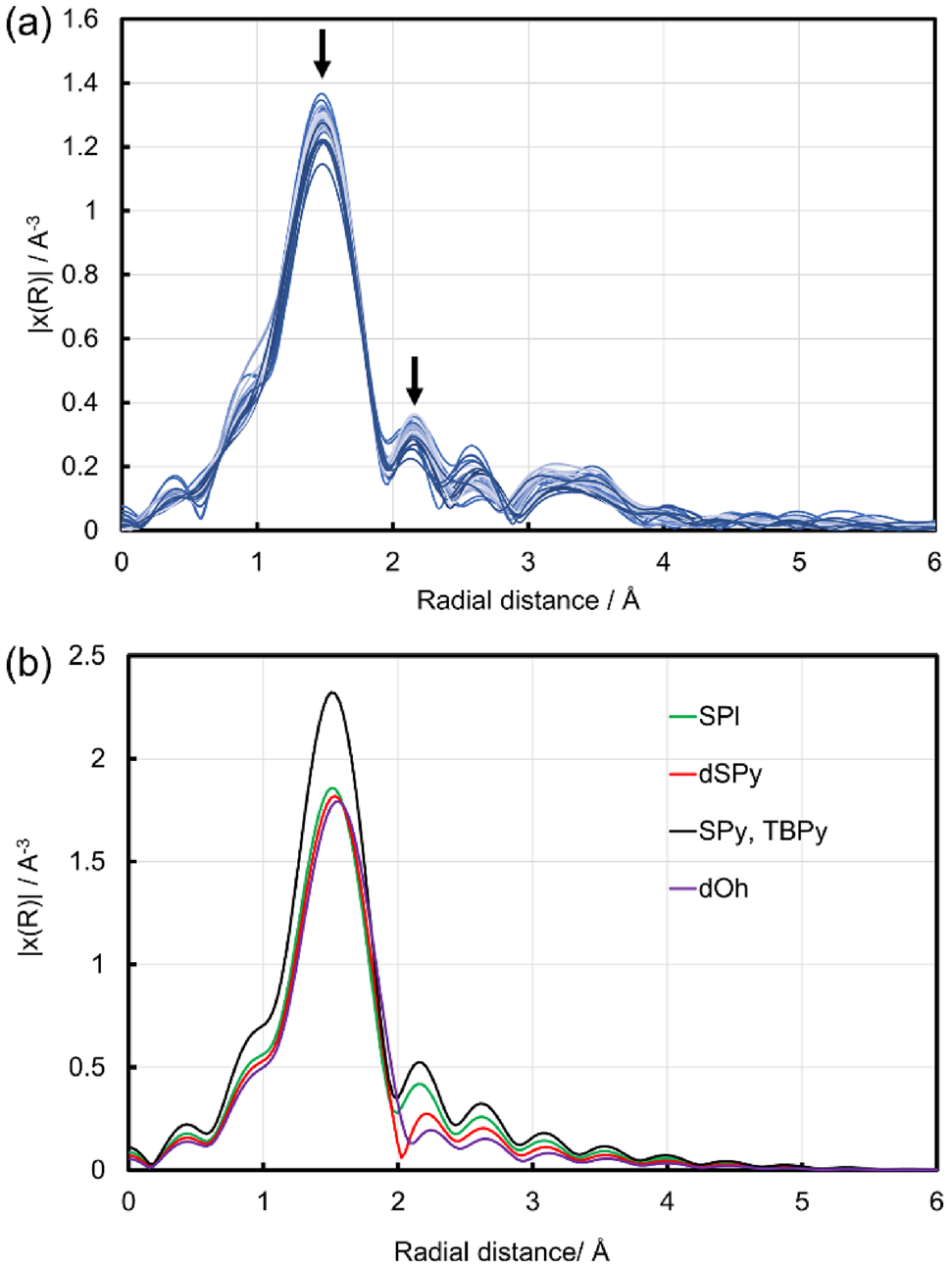
(**a**) Cu K-edge FT-EXAFS spectra for the twenty-eight Cu zeolites presented in Fig. 1. The two arrows indicate peaks at ca. 1.5 and 2.1 Å where the peak intensities are collected as structural variables of the Cu zeolites. (**b**) Simulated Cu K-edge FT-EXAFS spectra for four types of Cu local structures having 4–6 coordinated oxygen atoms as models of hydrated Cu^2+^ with dOh, dSPy, SPy, TBPy and SPl structures.

The data describing the Cu active site features are obtained by Cu K-edge XAFS spectral analysis, where the electronic state and local structure of the Cu species are evaluated. The Cu K-edge XANES spectra of all Cu zeolites listed in Fig. 1 are presented in Figs. 2 and S6 together with those for Cu_2_O, Cu(OH)_2_ and CuO powders as references of Cu^+^ and Cu^2+^. The XANES spectrum of Cu(NO_3_)_2_ aq is also taken as a reference of hydrated Cu^2+^. All Cu zeolites exhibit an X-ray absorption edge at similar energy to Cu(OH)_2_, CuO and Cu(NO_3_)_2_ aq, but at higher energy than Cu_2_O, indicating that all the Cu species in the zeolites are Cu^2+^. Furthermore, the Cu^2+^ species in the zeolites are attributable to hydrated Cu^2+^ because the spectra are similar to Cu(NO_3_)_2_ aq. However, the Cu zeolites exhibit slightly different X-ray absorption edge profiles from each other, suggesting different electronic state or coordination number/symmetry for the Cu species^29,51^. Thus, the edge energies at 0.5 of the normalized absorption are evaluated as a means to describe the structure of the Cu species. It should also be noted that the XAFS spectrum of a Cu zeolite is not changed by immersion in H_2_O (Fig. S7). The result suggests that the XAFS spectra of Cu zeolites (Figs. 2, 3) reflect the sample state in the liquid phase reaction conditions.

The local structure of the Cu species is evaluated from the Fourier transform (FT) of the EXAFS spectra. The Cu K-edge FT EXAFS spectra for all the Cu zeolites in Fig. 1 are presented in Figs. 3a and S8. The peak at ca. 1.5 Å is assignable to Cu–O scattering, which shows differences in the peak intensity between the Cu zeolites. In addition, the peak intensity at ca. 2.1 Å is also significantly different between the Cu zeolites. The spectral differences suggest a difference of local structure around the Cu species. In fact, previous studies on an aqueous solution of Cu^2+^ revealed that hydrated Cu^2+^ can have various local structures in dynamic equilibrium including: a distorted octahedron with six H_2_O coordinated Cu^2+^ (dOh), a distorted square pyramid (dSPy), a square pyramid (SPy), a regular trigonal bipyramid (TBPy) with five H_2_O coordinated ones and a square planner (SPl) structure with four H_2_O coordinated one^51^. Accordingly, the FT EXAFS spectra of the various hydrated Cu^2+^ species may be simulated by FEFF 6 calculations using simple local structure models of Cu^2+^(H_2_O)_x_ (x = 4–6), where the two kinds of single scattering of Cu–O with bond lengths of 1.96 Å and 2.40 Å are calculated based on the literature, and summed up to construct each model structure^47,51^. The structural parameters for each model structure are shown in Table S4. The simulated FT EXAFS spectra are presented in Fig. 3b and show that the peak intensities at ca. 1.5 and 2.1 Å vary with the local structure. Therefore, the peak intensities at ca. 1.5 and 2.1 Å are extracted as descriptors of the local structure of Cu.

**Figure 4:**
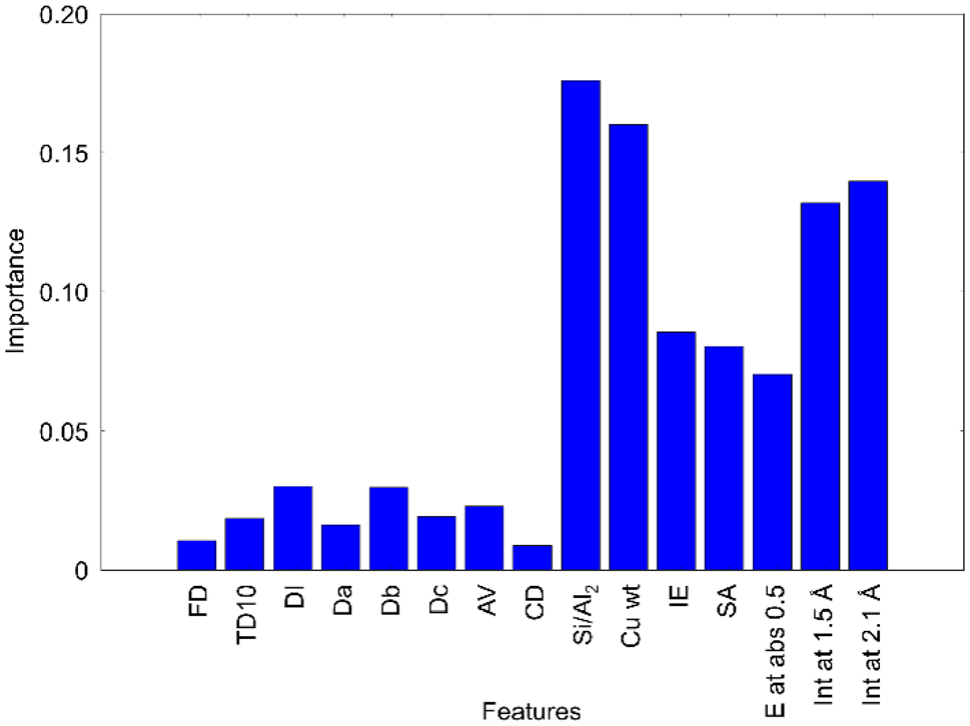
Importance of explanatory variables evaluated by the random forest classification.

The values of the 15 descriptors of the twenty-eight Cu zeolite catalysts are listed in Table S5 together with the specific activity. To explore the important descriptors for the specific activity, the random forest classification method is deployed for the data of the twenty-eight Cu zeolites. As a data pretreatment, the specific activity is classified into three groups, i.e., low, medium and high using Gaussian Mixture model within unsupervised machine learning in order to perform random forest classification. The classified specific activity is listed in Table S5. Here, the explanatory variables are set to fifteen descriptors of the Cu zeolite catalysts while the objective variable is set to the classified specific activity. Then, the trained random forest classification with the 15 descriptors is evaluated by cross-validation, which returns an average score of 68%. The importance of each descriptor is evaluated and the results are presented in Fig. 4. Relatively high importance is assigned to seven variables including Si/Al_2_, Cu wt, IE, SA, E at abs 0.5, Int at 1.5 Å and Int at 2.1 Å, which are the structural parameters or compositions of the Cu zeolites. In contrast, the descriptors of zeolite types and pores including FD, TD10, DI, Da-c, AV and CD have less impact on the specific activity. It is suggested that the Cu zeolite structure and/or composition are the key descriptors of catalytic activity.

**Figure 5:**
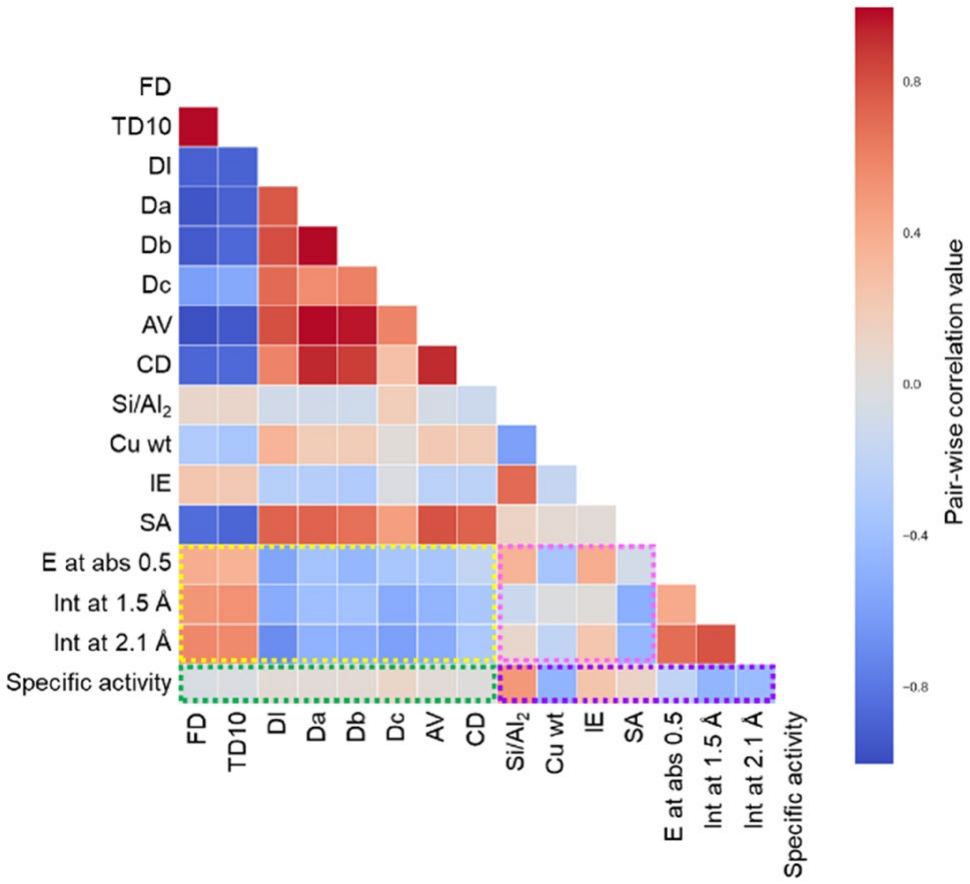
Heat map for pairwise correlation between structural variables and specific activity.

Pairwise correlations of the 16 variables including both explanatory variables and objective one are evaluated in terms of the Pearson correlation coefficient and the results are presented in Fig. 5. The dark color in red or blue means high positive or high negative correlation coefficients, respectively, which suggest a strong correlation between the pair variables. Accordingly, the correlations between the specific activity and the seven variables from FD to CD representing zeolite framework structure are weak (See the green square in Fig. 5). However, there are relatively strong correlations between the specific activity and the other seven variables from Si/Al_2_ to the Int at 2.1 Å (the purple square in Fig. 5). Note that the strength of the pairwise correlation is consistent with the importance of the random forest classification. Thus, both data analyses suggest that the seven variables relating to Cu zeolite structure and composition are important descriptors of the specific activity. Interestingly, relatively strong correlations are observed for the pairs between the variables of the zeolite framework structure (from FD to CD) and those of the Cu structure (E at abs 0.5, Int at 1.5 and 2.1 Å) (the yellow square in Fig. 5), suggesting that the zeolite framework structure affects the structure of the Cu active site. In addition, significant correlations are also found among the pairs between the variables from Si/Al_2_ to SA and those from E at abs 0.5 to Int at 2.1 Å (the pink square in Fig. 5). Thus, one can consider that the zeolite framework structure, composition and surface area determine the structure of the Cu active site, which strongly affects the catalytic activity.

**Figure 6:**
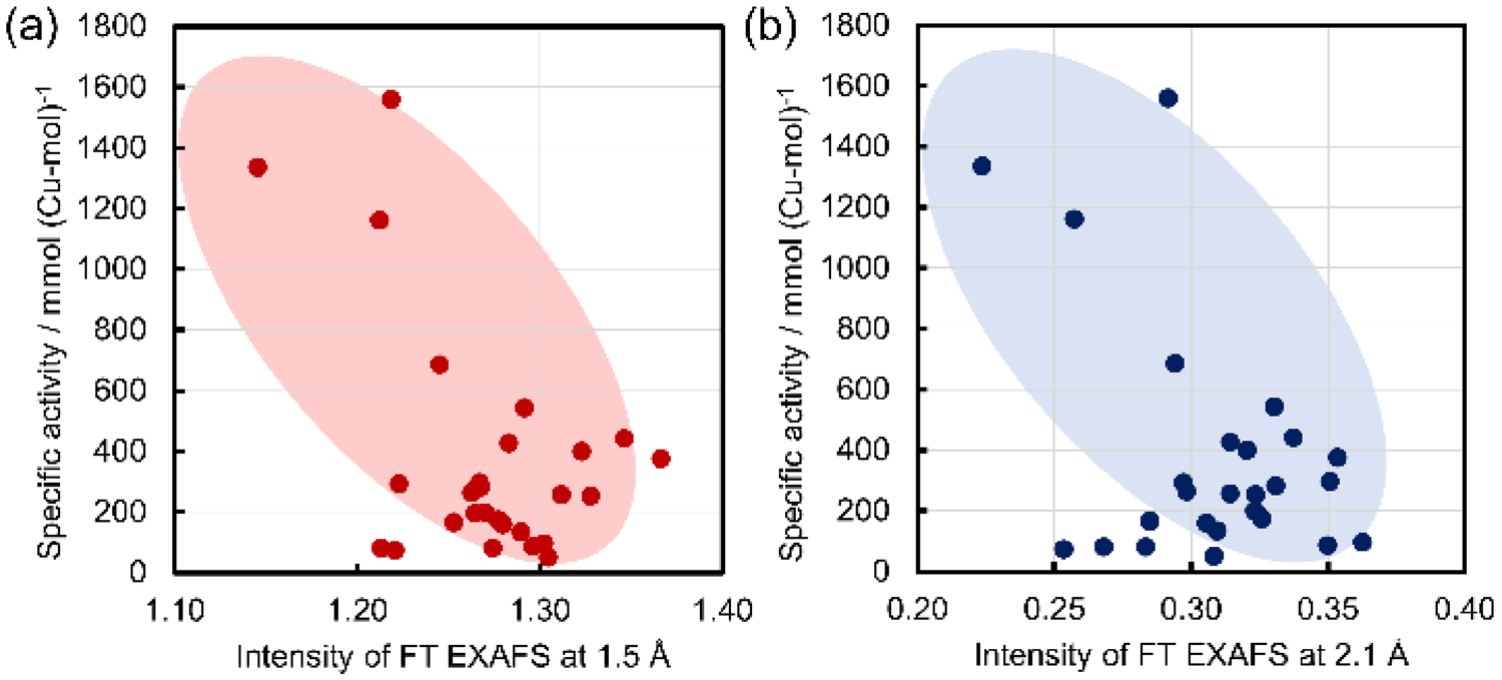
Plots of the specific activity against (**a**) the intensities of FT EXAFS at 1.5 Å and (**b**) the intensities of FT EXAFS at 2.1 Å.

**Figure 7:**
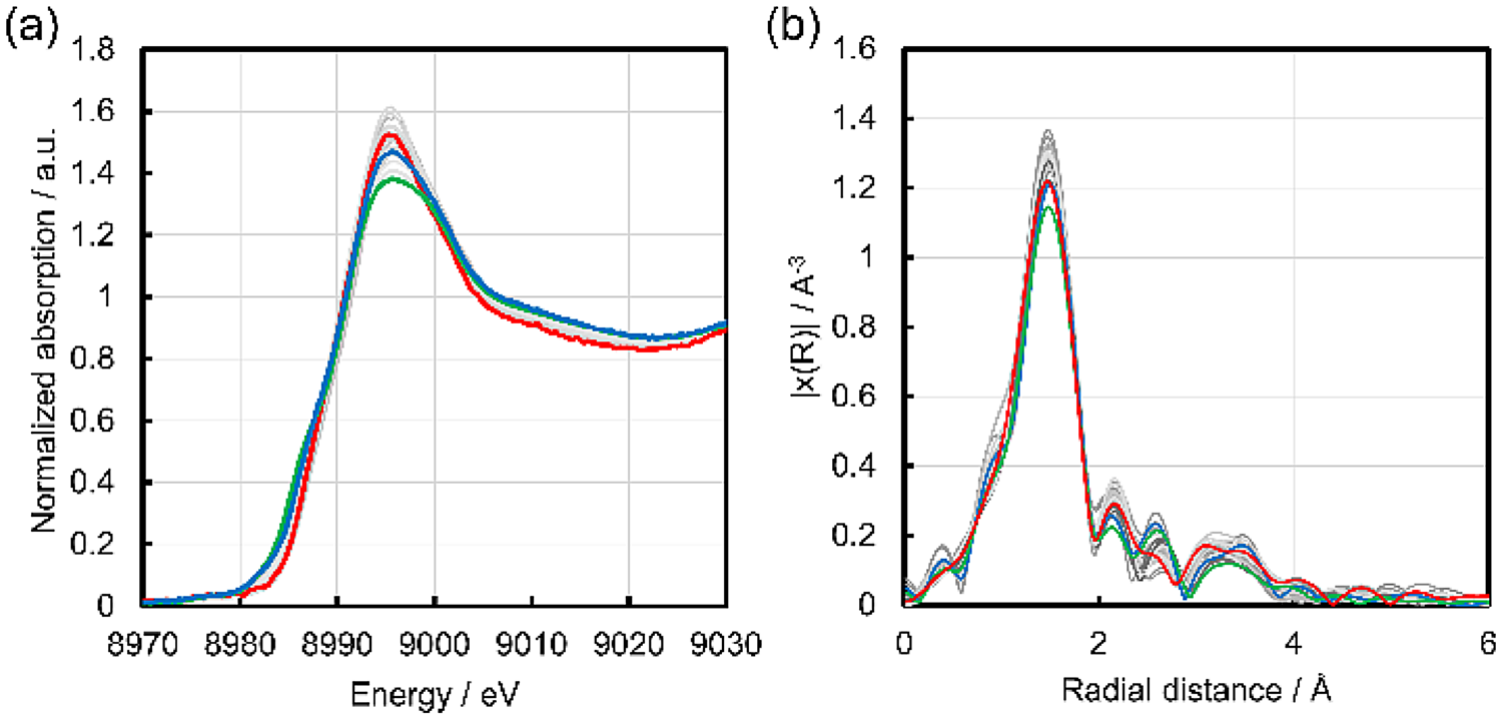
Cu K-edge XANES and FT-EXAFS spectra for Cu(0.64)FAU(14.9) (green), Cu(0.64)FAU(110) (blue), Cu(0.40)MOR(220) (red), and the other Cu zeolites (gray).

In order to specify a highly active Cu structure, the specific activity is plotted against the intensities of FT-EXAFS at 1.5 and 2.1 Å as shown in Fig. 6. In both cases, the specific activity increases with the decrease of the intensities of the FT EXAFS. Note that the SPy and TBPy structures have five Cu–O bonds of length 1.96 Å, which show the highest intensity at 1.5 and 2.1 Å among the simulated spectra (Fig. 6). Thus, the SPy and TBPy structures are not highly active species. In other words, any of the other structures dOh, dSPy and SPl have high specific activity. Given that the Cu species in zeolites are considered mixtures of various structures, the main structures are difficult to be determined only from the intensities of FT EXAFS. In addition, the Int at 2.1 Å might be affected significantly by noise in the EXAFS spectra (Fig. S9). Thus, the Cu K-edge XANES spectral features of the active Cu zeolites are also examined for further specification of the active structure, because the XANES feature is sensitive to the local structure and is less affected by the noise than the EXAFS. Figure 7 displays the XANES spectra of Cu(0.40)MOR(220), Cu(0.64)FAU(110) and Cu(1.11)FAU(14.9) with a high specific activity. The spectra of the two CuFAU catalysts have a shoulder at 8986 eV and have a relatively low white line intensity at 8995 eV, features which are similar to the SPl structure reported in the literature^29^. In particular, the shoulder at 8986 eV is assignable to the electronic transition 1s to 4p of the Cu^2+^ species with the SPl structure. Therefore, the SPl structure is considered to be the highly active structure in FAU. In the case of Cu(0.40)MOR(220), the XANES spectrum does not show such a shoulder at 8986 eV but shows a relatively high white line intensity. Such a spectral feature is seen in dOh and dSPy. In addition, the FT-EXAFS of Cu(0.40)MOR(220) does not exhibit a peak shift at 1.5 Å as is the case for the two CuFAU with SPl structures, suggesting that the dSPy structure is formed in Cu(0.40)MOR(220), because dOh should show the peak shift as simulated in Fig. 4. Therefore, the dSPy structure is proposed as the highly active structure in MOR.

## Conclusions

Various metal zeolites are prepared and tested for direct oxidation of CH_4_ to CH_3_OH using H_2_O_2_ as an oxidant. Given that Cu is effective for the selective oxidation to CH_3_OH and CH_3_OOH without producing HCOOH, the catalytic performance of 35 Cu zeolites and 20 H zeolites having MOR, FAU, BEA, FER, CHA, and MFI frameworks are evaluated, where the Cu zeolites except for Cu-MFI are confirmed to show catalytic activity for the CH_4_–H_2_O_2_ reaction. In addition, the CuMOR and CuFAU zeolites contain highly active Cu species among the Cu zeolites tested. The catalytically active twenty-eight Cu zeolites are described in terms of the structural variables of the zeolite framework obtained from the database of the IZA and the experimentally evaluated zeolite features based on composition, surface area and local structure of the Cu active site. The relationships between the specific activity of the Cu zeolites and the structural variables are analyzed by classification methods using unsupervised and supervised machine learning and by pairwise correlation, suggesting that the local structure of the Cu active species, represented by the intensities of FT-EXAFS at 1.5 and 2.1 Å are the important descriptors for the specific activity. By comparing the experimental XAFS spectra with the simulated or reported ones, highly active Cu species in FAU and MOR are considered to have SPl and dSPy structures, respectively.

## Supplementary Information

Supplementary Information.

## References

[1] Ross MO (2019). Particulate methane monooxygenase contains only mononuclear copper centers. Science.

[2] Haruta M (1997). Size- and support-dependency in the catalysis of gold. Catal. Today.

[3] Behrens M (2012). The active site of methanol synthesis over Cu/ZnO/Al_2_O_3_ industrial catalysts. Science.

[4] Murata K (2017). The metal-support interaction concerning the particle size effect of Pd/Al_2_O_3_ on methane combustion. Angew. Chem. Int. Ed..

[5] Snyder BER, Bols ML, Schoonheydt RA, Sels BF, Solomon EI (2018). Iron and copper active sites in zeolites and their correlation to metalloenzymes. Chem. Rev..

[6] Takahashi K (2019). The rise of catalyst informatics: Towards catalyst genomics. ChemCatChem.

[7] Goldsmith BR, Esterhuizen J, Liu J-X, Bartel CJ, Sutton C (2018). Machine learning for heterogeneous catalyst design and discovery. AlChE J..

[8] Klanner C (2004). The development of descriptors for solids: Teaching “catalytic intuition” to a computer. Angew. Chem. Int. Ed..

[9] Hattori T, Kito S (1995). Neural-network as a tool for catalyst development. Catal. Today.

[10] Sirajuddin S, Rosenzweig AC (2015). Enzymatic oxidation of methane. Biochemistry.

[11] Mahyuddin MH, Shiota Y, Staykov A, Yoshizawa K (2018). Theoretical overview of methane hydroxylation by copper–oxygen species in enzymatic and zeolitic catalysts. Acc. Chem. Res..

[12] Cao L, Caldararu O, Rosenzweig AC, Ryde U (2018). Quantum refinement does not support dinuclear copper sites in crystal structures of particulate methane monooxygenase. Angew. Chem. Int. Ed..

[13] Groothaert MH, Smeets PJ, Sels BF, Jacobs PA, Schoonheydt RA (2005). Selective oxidation of methane by the bis(μ-oxo)dicopper core stabilized on ZSM-5 and mordenite zeolites. J. Am. Chem. Soc..

[14] Grundner S (2015). Single-site trinuclear copper oxygen clusters in mordenite for selective conversion of methane to methanol. Nat. Commun..

[15] Lange J-P, Sushkevich VL, Knorpp AJ, van Bokhoven JA (2019). Methane-to-methanol via chemical looping: Economic potential and guidance for future research. Ind. Eng. Chem. Res.

[16] Hammond C (2012). Direct catalytic conversion of methane to methanol in an aqueous medium by using copper-promoted Fe-ZSM-5. Angew. Chem. Int. Ed..

[17] Xiao P, Wang Y, Nishitoba T, Kondo JN, Yokoi T (2019). Selective oxidation of methane to methanol with H_2_O_2_ over an Fe-MFI zeolite catalyst using sulfolane solvent. Chem. Commun..

[18] Sheppard T, Hamill CD, Goguet A, Rooney DW, Thompson JM (2014). A low temperature, isothermal gas-phase system for conversion of methane to methanol over Cu-ZSM-5. Chem. Commun..

[19] Ipek B, Lobo RF (2016). Catalytic conversion of methane to methanol on Cu-SSZ-13 using N_2_O as oxidant. Chem. Commun..

[20] Alayon EM, Nachtegaal M, Ranocchiari M, van Bokhoven JA (2012). Catalytic conversion of methane to methanol over Cu-mordenite. Chem. Commun..

[21] Narsimhan K, Iyoki K, Dinh K, Román-Leshkov Y (2016). Catalytic oxidation of methane into methanol over copper-exchanged zeolites with oxygen at low temperature. ACS Cent. Sci..

[22] Sushkevich VL, Palagin D, Ranocchiari M, van Bokhoven JA (2017). Selective anaerobic oxidation of methane enables direct synthesis of methanol. Science.

[23] Hammond C (2013). Elucidation and evolution of the active component within Cu/Fe/ZSM-5 for catalytic methane oxidation: From synthesis to catalysis. ACS Catal..

[24] Hammond C (2012). Catalytic and mechanistic insights of the low-temperature selective oxidation of methane over Cu-promoted Fe-ZSM-5. Chem. Euro. J..

[25] Tomkins P, Ranocchiari M, van Bokhoven JA (2017). Direct conversion of methane to methanol under mild conditions over Cu-zeolites and beyond. Acc. Chem. Res..

[26] Tomkins P (2016). Isothermal cyclic conversion of methane into methanol over copper-exchanged zeolite at low temperature. Angew. Chem. Int. Ed..

[27] Parfenov MV, Starokon EV, Pirutko LV, Panov GI (2014). Quasicatalytic and catalytic oxidation of methane to methanol by nitrous oxide over FeZSM-5 zeolite. J. Catal..

[28] Pappas DK (2017). Methane to methanol: Structure-activity relationships for Cu-CHA. J. Am. Chem. Soc..

[29] Martini A (2017). Composition-driven Cu-speciation and reducibility in Cu-CHA zeolite catalysts: A multivariate XAS/FTIR approach to complexity. Chem. Sci..

[30] Shichi A, Satsuma A, Hattori T (2001). Influence of geometry-limited diffusion on the selective catalytic reduction of NO by hydrocarbons over Cu-exchanged zeolite. Appl. Catal. B.

[31] Satsuma, A., Iwase, M., Shichi, A., Hattori, T. & Murakami, Y. Factors controlling catalytic activity of H-form zeolites for the selective reduction of NO with CH_4_. in *Stud. Surf. Sci. Catal.* Vol. 105 (eds Chon, H., Ihm, S.-K. & Uh, Y. S.) 1533–1540 (Elsevier, 1997).

[32] Takahashi K, Miyazato I, Nishimura S, Ohyama J (2018). Unveiling hidden catalysts for the oxidative coupling of methane based on combining machine learning with literature data. ChemCatChem.

[33] Ohyama J, Nishimura S, Takahashi K (2019). Data driven determination of reaction conditions in oxidative coupling of methane via machine learning. ChemCatChem.

[34] Miyazato I, Nishimura S, Takahashi L, Ohyama J, Takahashi K (2020). Data-driven identification of the reaction network in oxidative coupling of the methane reaction via experimental data. J. Phys. Chem. Lett..

[35] Toyao T (2020). Machine learning for catalysis informatics: Recent applications and prospects. ACS Catal..

[36] Meyer B, Sawatlon B, Heinen S, von Lilienfeld OA, Corminboeuf C (2018). Machine learning meets volcano plots: Computational discovery of cross-coupling catalysts. Chem. Sci..

[37] Kitchin JR (2018). Machine learning in catalysis. Nat. Catal..

[38] Muraoka K, Sada Y, Miyazaki D, Chaikittisilp W, Okubo T (2019). Linking synthesis and structure descriptors from a large collection of synthetic records of zeolite materials. Nat. Commun..

[39] Nguyen TN (2020). High-throughput experimentation and catalyst informatics for oxidative coupling of methane. ACS Catal..

[40] Daeyaert F, Ye F, Deem MW (2019). Machine-learning approach to the design of OSDAs for zeolite beta. Proc. Natl. Acad. Sci..

[41] Jensen Z (2019). A machine learning approach to zeolite synthesis enabled by automatic literature data extraction. ACS Central Sci..

[42] Baerlocher, C. & McCusker, L. B. *Database of Zeolite Structures*. http://www.iza-structure.org/databases/. Accessed 16 May 2019.

[43] Ueda K, Ohyama J, Satsuma A (2017). In situ XAFS study of dynamic behavior of Cu species in MFI-zeolite under element gases of ammonia selective catalytic reduction. Chem. Lett..

[44] Frahm R (1989). New method for time dependent x-ray absorption studies. Rev. Sci. Instrum..

[45] Sheppard T, Daly H, Goguet A, Thompson JM (2016). Improved efficiency for partial oxidation of methane by controlled copper deposition on surface-modified ZSM-5. ChemCatChem.

[46] Ravel B, Newville M (2005). ATHENA, ARTEMIS, HEPHAESTUS: Data analysis for X-ray absorption spectroscopy using IFEFFIT. J. Synchrotron. Radiat..

[47] Zabinsky SI, Rehr JJ, Ankudinov A, Albers RC, Eller MJ (1995). Multiple-scattering calculations of x-ray-absorption spectra. Phys. Rev. B.

[48] Pedregosa F (2011). Scikit-learn: Machine learning in python. J. Mach. Learn. Res..

[49] Breiman L (2001). Random forests. Mach. Learn..

[50] Osadchii DY (2018). Isolated Fe sites in metal organic frameworks catalyze the direct conversion of methane to methanol. ACS Catal..

[51] Chaboy J, Muñoz-Páez A, Merkling PJ, Marcos ES (2006). The hydration of Cu2+: Can the Jahn-Teller effect be detected in liquid solution?. J. Chem. Phys..

