# Supplementary material for: Data science assisted investigation of catalytically active copper hydrate in zeolites for direct oxidation of methane to methanol using H2O2
Source: Sci Rep. 2021 Jan 22;11:2067. doi: 10.1038/s41598-021-81403-4 (PMC7822835; doi:10.1038/s41598-021-81403-4)
Supplement: Supplementary file 1 — Supplementary Information. [file 41598_2021_81403_MOESM1_ESM.docx]

***Supplementary Information***

**Data Science Assisted Investigation of Catalytically Active Copper Hydrate in Zeolites for Direct Oxidation of Methane to Methanol Using H_2_O_2_**

Junya Ohyama,^a,*^ Airi Hirayama,^b^ Nahoko Kondou,^a^ Hiroshi Yoshida,^a^ Masato Machida,^a^ Shun Nishimura,^c^ Kenji Hirai,^d^ Itsuki Miyazato,^e^ Keisuke Takahashi^e^

^a^ Faculty of Advanced Science and Technology, Kumamoto University, 2-39-1 Kurokami, Chuo-ku, Kumamoto, 860-8555 Japan

^b^ Department of Applied Chemistry and Biochemistry, Graduate School of Science and Technology, Kumamoto University, 2-39-1 Kurokami, Chuo-ku, Kumamoto, 860-8555, Japan

^c^ Graduate School of Advanced Science and Technology, Japan Advanced Institute of Science and Technology (JAIST), 1-1 Asahidai, Nomi, 923-1292 Japan

^d^ Research Institute for Electronic Science, Hokkaido University, N20 W10, Kita-Ward, 001-0020 Japan

^e^ Department of Chemistry, Hokkaido University, N-15 W-8, Sapporo 060-0815, Japan

* ohyama@kumamoto-u.ac.jp


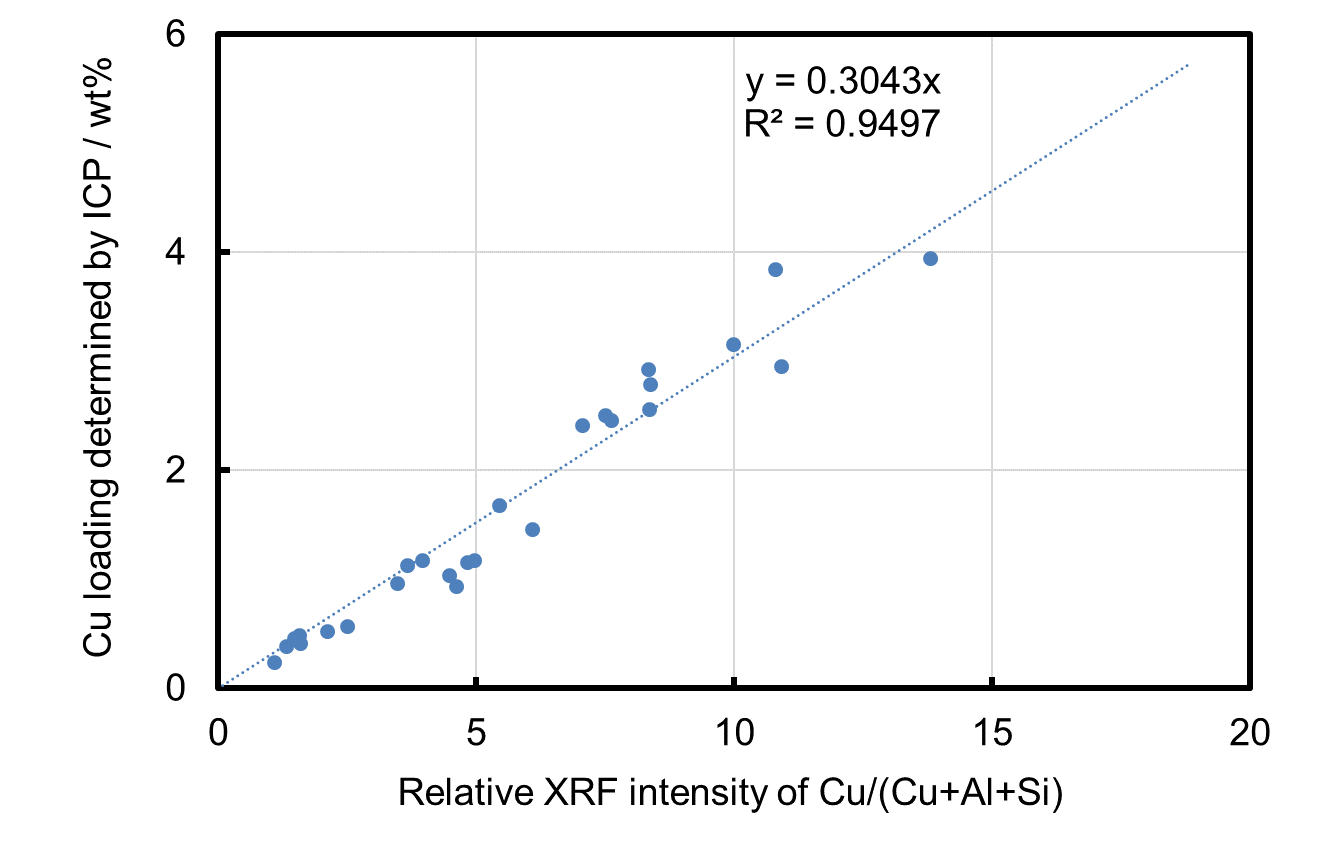


**Figure S1** Relationship between the relative XRF intensity of Cu/(Cu+Al+Si) and the Cu loading determined by ICP-OES.


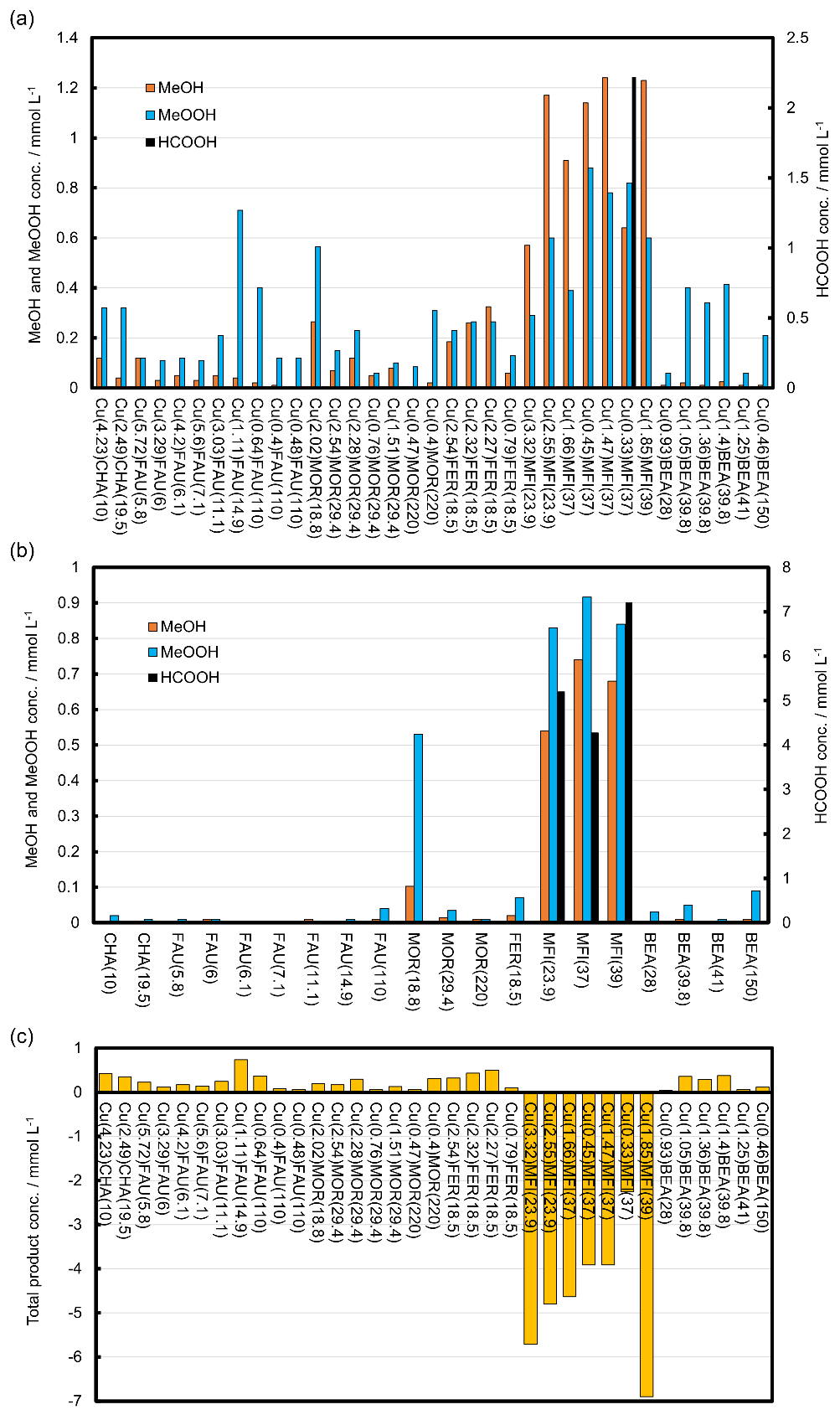


**Figure S2.** Results for the CH_4_-H_2_O_2_ reaction using (a) thirty-five Cu zeolites and (b) twenty H zeolites. Reaction conditions: CH_4_ 3.5 MPa, 30 wt% H_2_O_2_ 155 μl, H_2_O 3 ml, catalyst 10 mg, 60°C, 1 h. The standard deviation of reaction result using Cu(2.02)-MOR(18.8) for four times is within 10% of the product amounts. (c) The product increments of Cu zeolites from H-zeolites, i.e., the difference between (a) and (b).


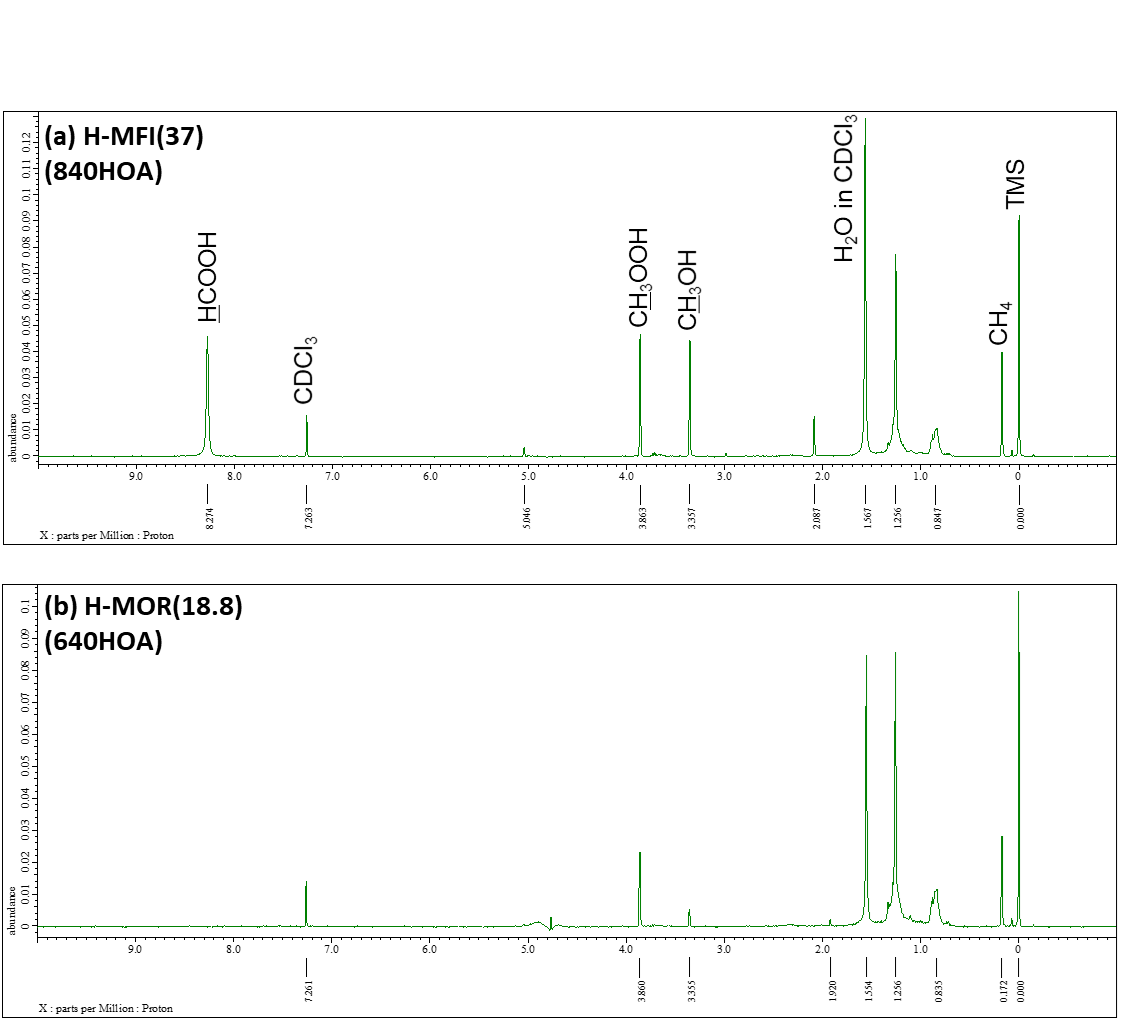


**
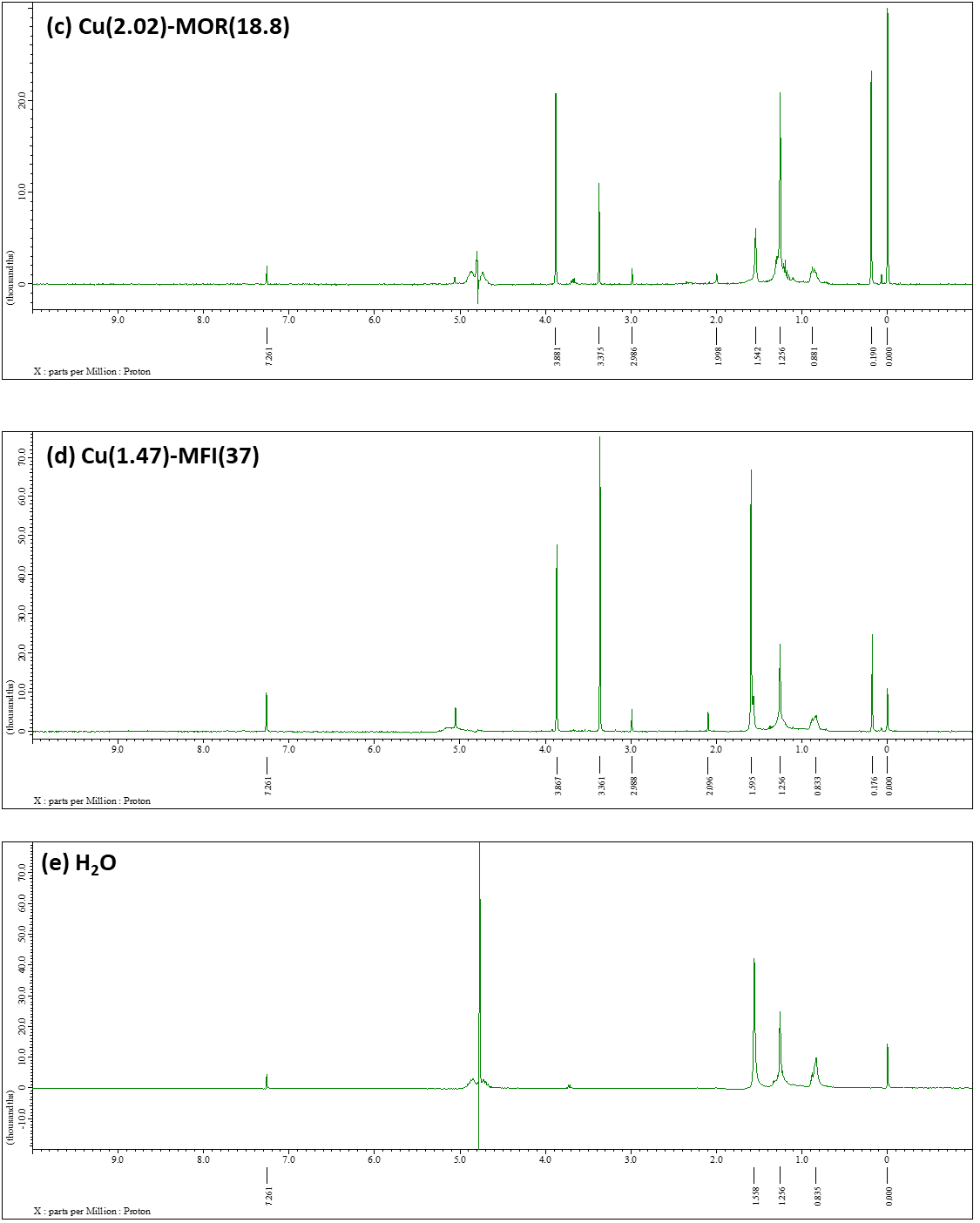
**

**
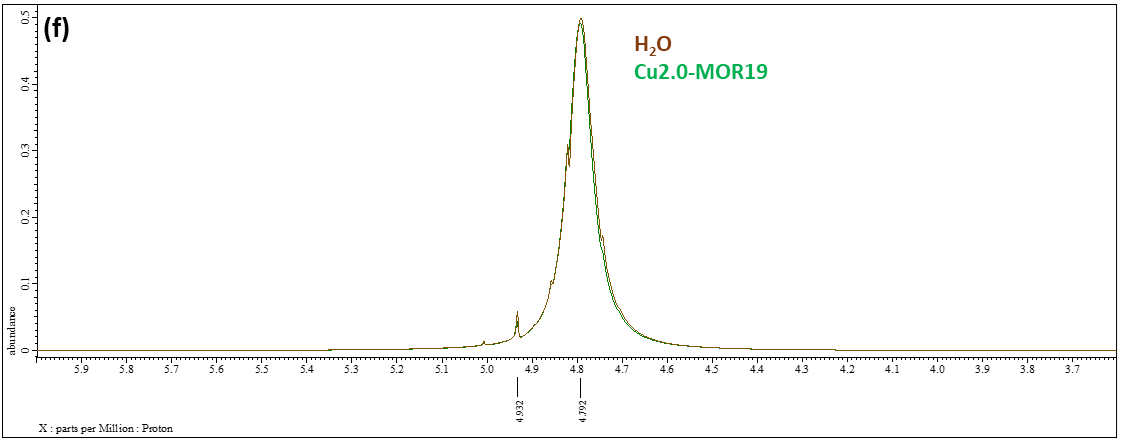
**

**Figure S3** ^1^H NMR spectra of the reaction solutions using (a) H-MFI(37), (b) H-MOR(18.8), (c) Cu(2.02)-MOR(18.8), (d) Cu(1.47)-MFI(37), and (e) H_2_O obtained with a water suppression technique. (f) ^1^H NMR spectra of the reaction solution using a Cu-MOR zeolite and a H_2_O-D_2_O solvent obtained without a water suppression technique as a reference. The small peaks at 2 and 3 ppm in (a)-(d) might be assignable to byproducts; however, they are small and considered not to affect the catalyst classification by unsupervised learning. The peaks at 1.26 and 0.84 ppm in (a)-(e) are assignable to impurities of solvents, and do not increase by the reaction. The peak at 4.9 ppm in (f) is also considered derived from impurity of H_2_O and/or D_2_O, and do not increased by the reaction.


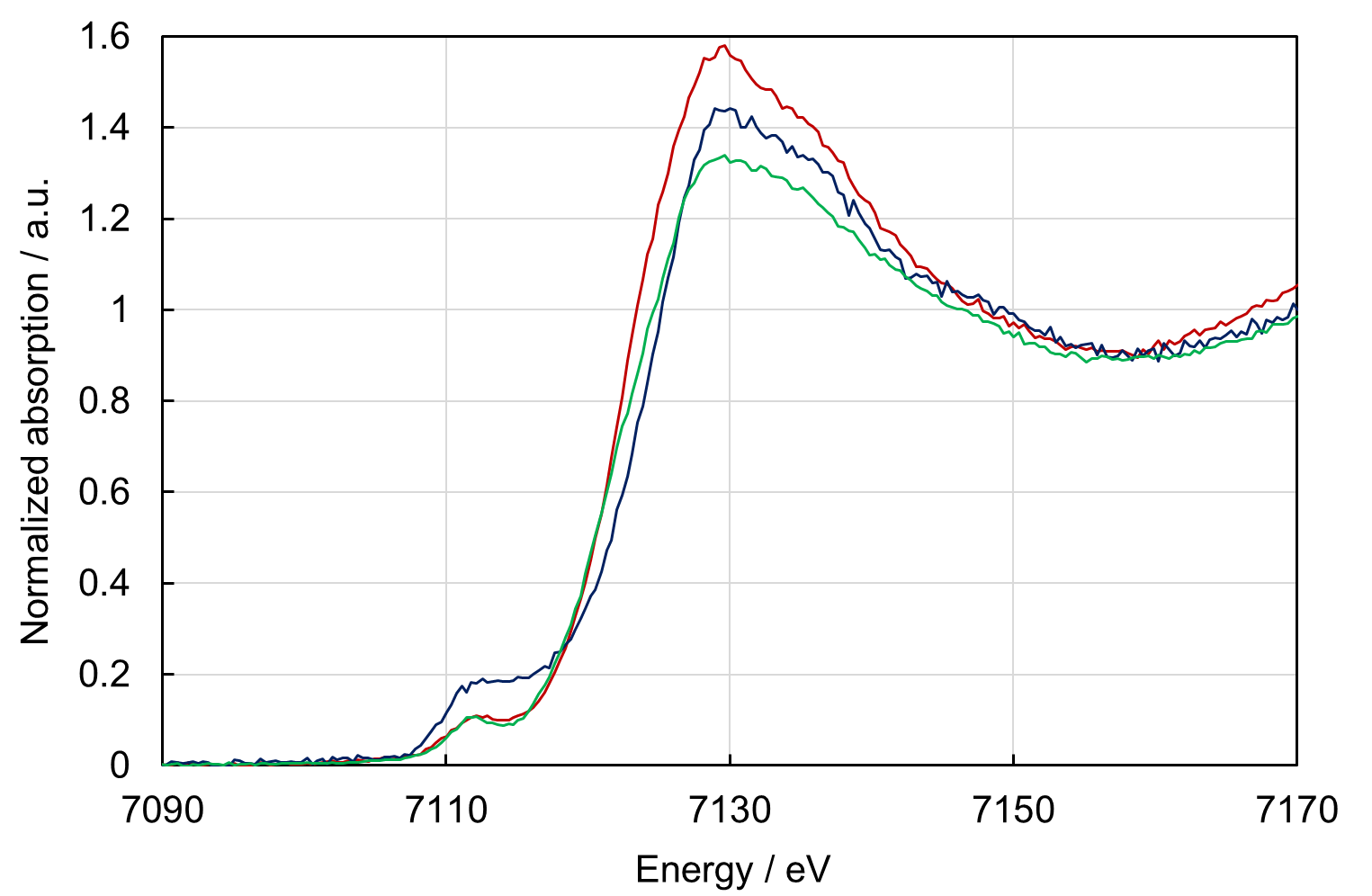


**Figure S4** Fe K edge XANES spectra of H-MFI(37) (red), H-MOR(29.4) (blue), and H-FAU(14.9) (green) obtained in a fluorescence mode using a Si drift detector at BL11 of SAGA Light Source. The different spectra suggest that the H-zeolites contain Fe species with different structures.


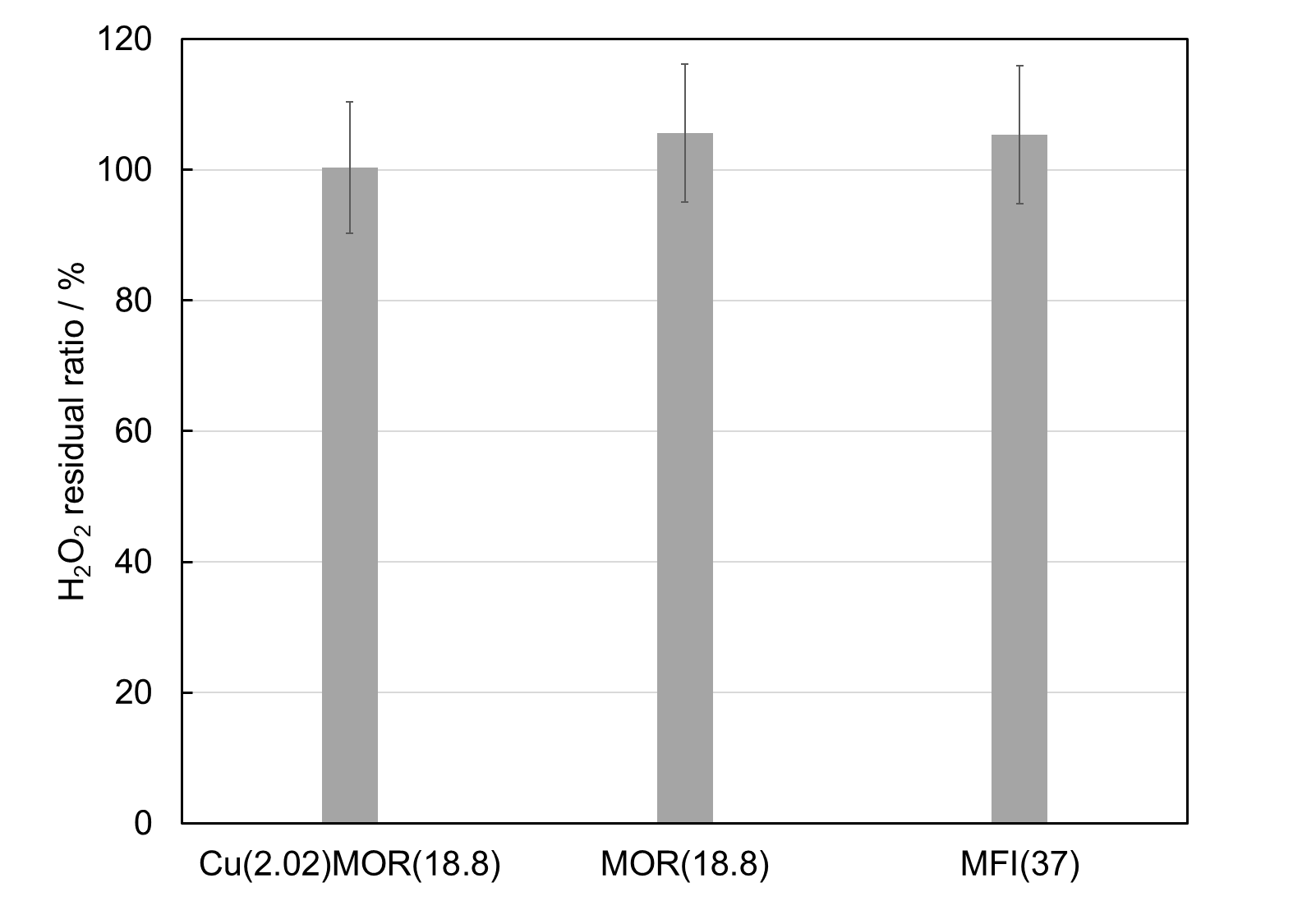


**Figure S5.** H_2_O_2_ residual ratio after CH_4_-H_2_O_2_ reaction using Cu(2.02)MOR(18.8), MOR(18.8), and MFI(37). The H_2_O_2_ concentration is determined by the I^3-^ method (Klassen, N.; Marchington, D.; McGowan, H., *Anal. Chem.* **1994**, *66*, 2921.): The following three solutions are prepared. Solution A: 33 g of KI, 1 g of NaOH, and 0.1 g of (NH_4_)_6_Mo_7_O_24_ are dissolved in 500 mL of H_2_O, and then save in the dark. Solution B: 10 g of potassium hydrogen phthalate is dissolved in 500 mL of H_2_O. Solution C: Diluted reaction solution (1/4000) after filtration. The above solutions are mixed at A:B:C=1:1:1, and the UV-vis spectrum is taken on a Thermo Scientific GENESYS 10S UV-Vis Spectrophotometer. The hydrogen peroxide concentration is calculated from the absorbance at 352 nm.


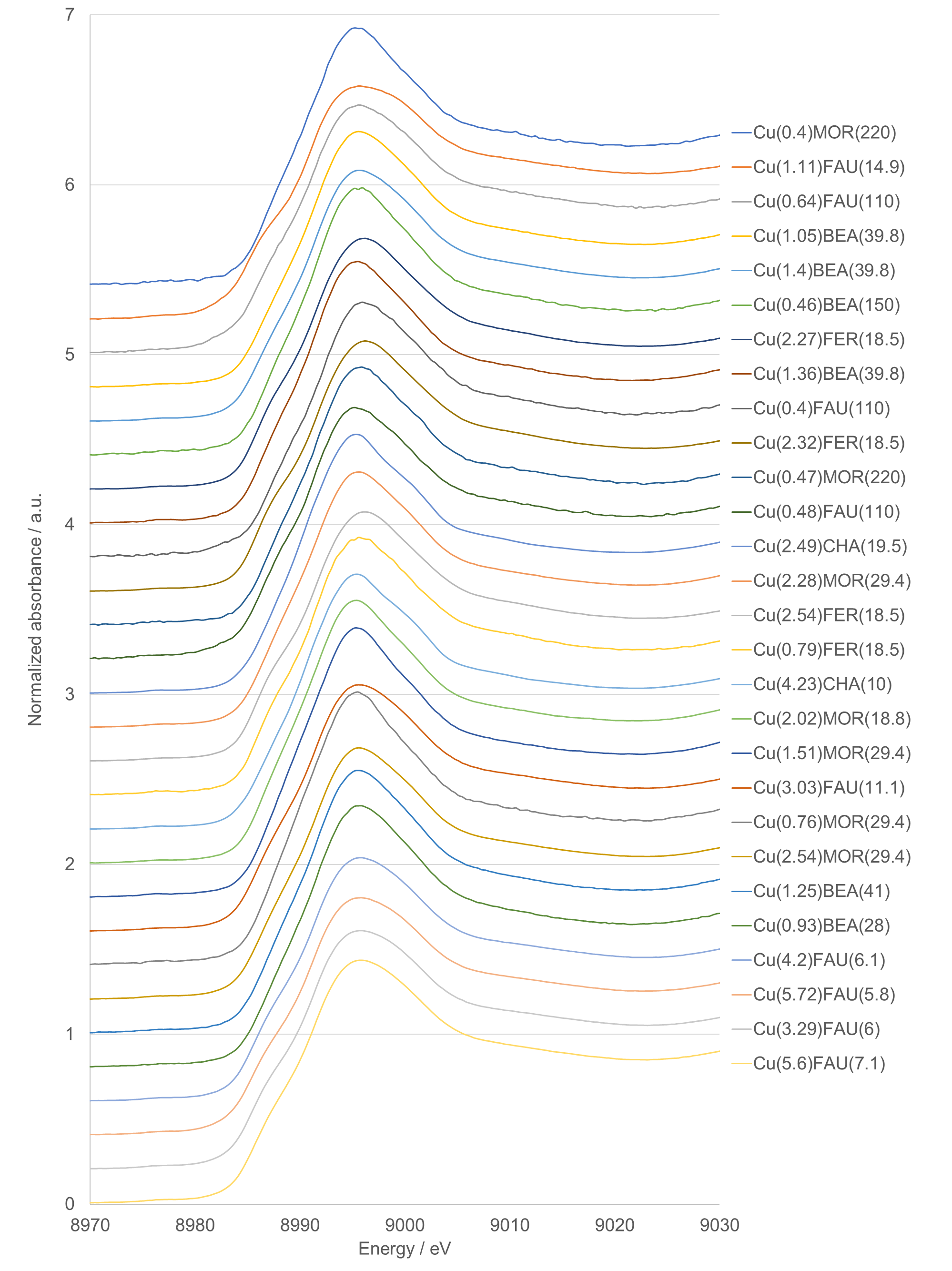


**Figure S6** Cu K edge XANES spectra for all Cu zeolites in Figure 1(d).


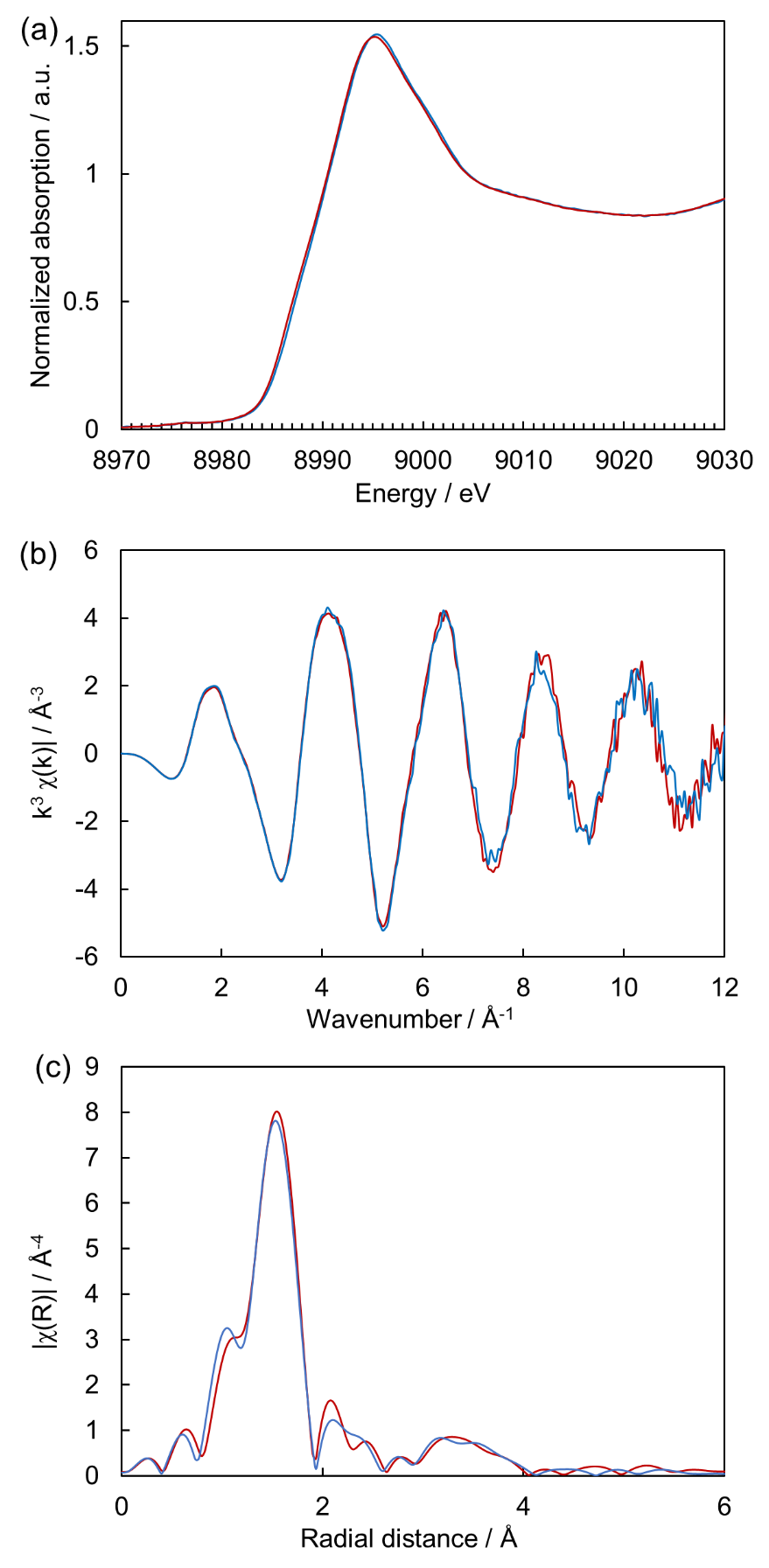


**Figure S7** Cu K edge (a) XANES, (b) EXAFS, and (c) FT-EXAFS spectra of Cu(2.02)-MOR(18.8) before (red) and after immersed in water (blue).


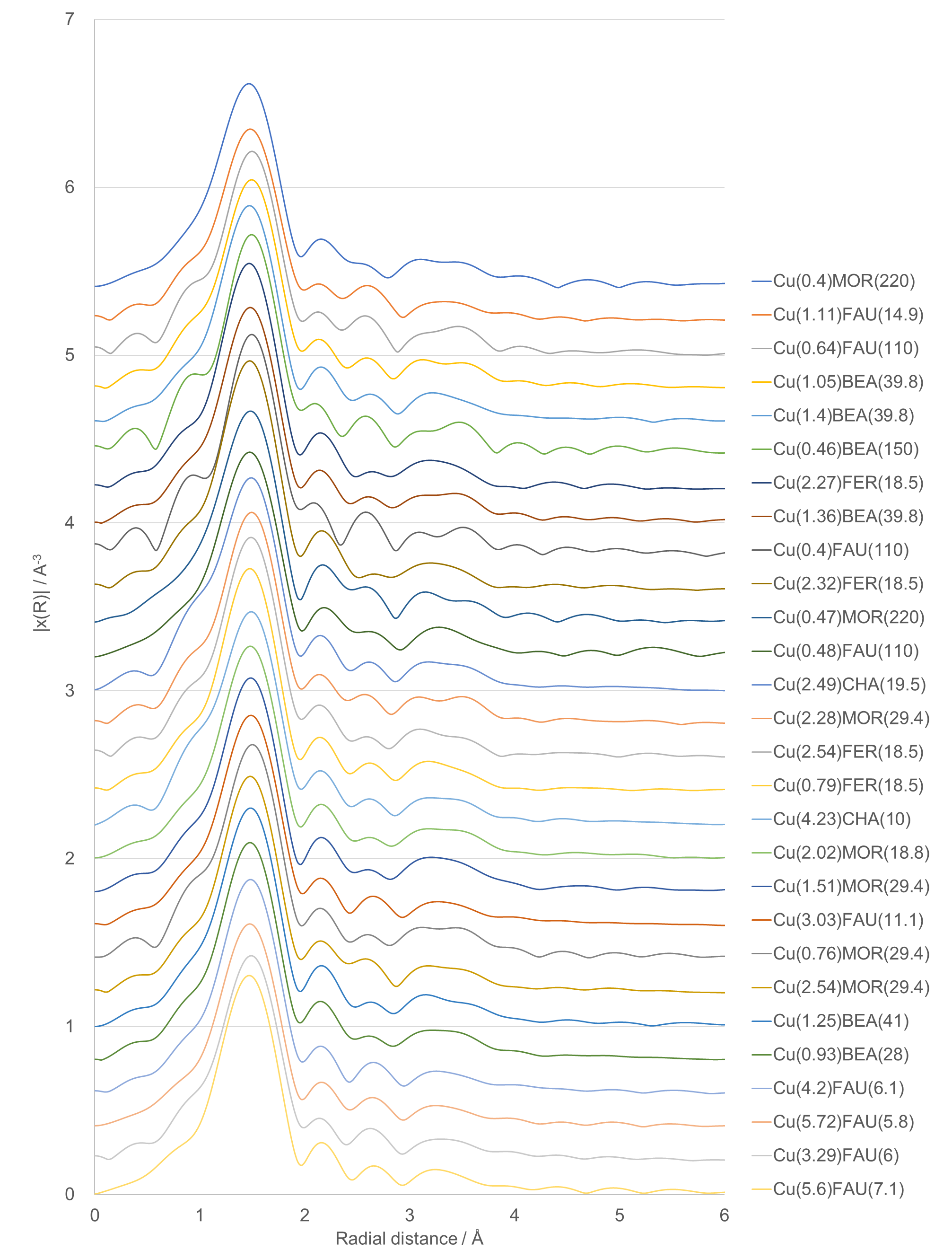


**Figure S8** Cu K edge FT EXAFS spectra for all Cu zeolites in Figure 1(d).


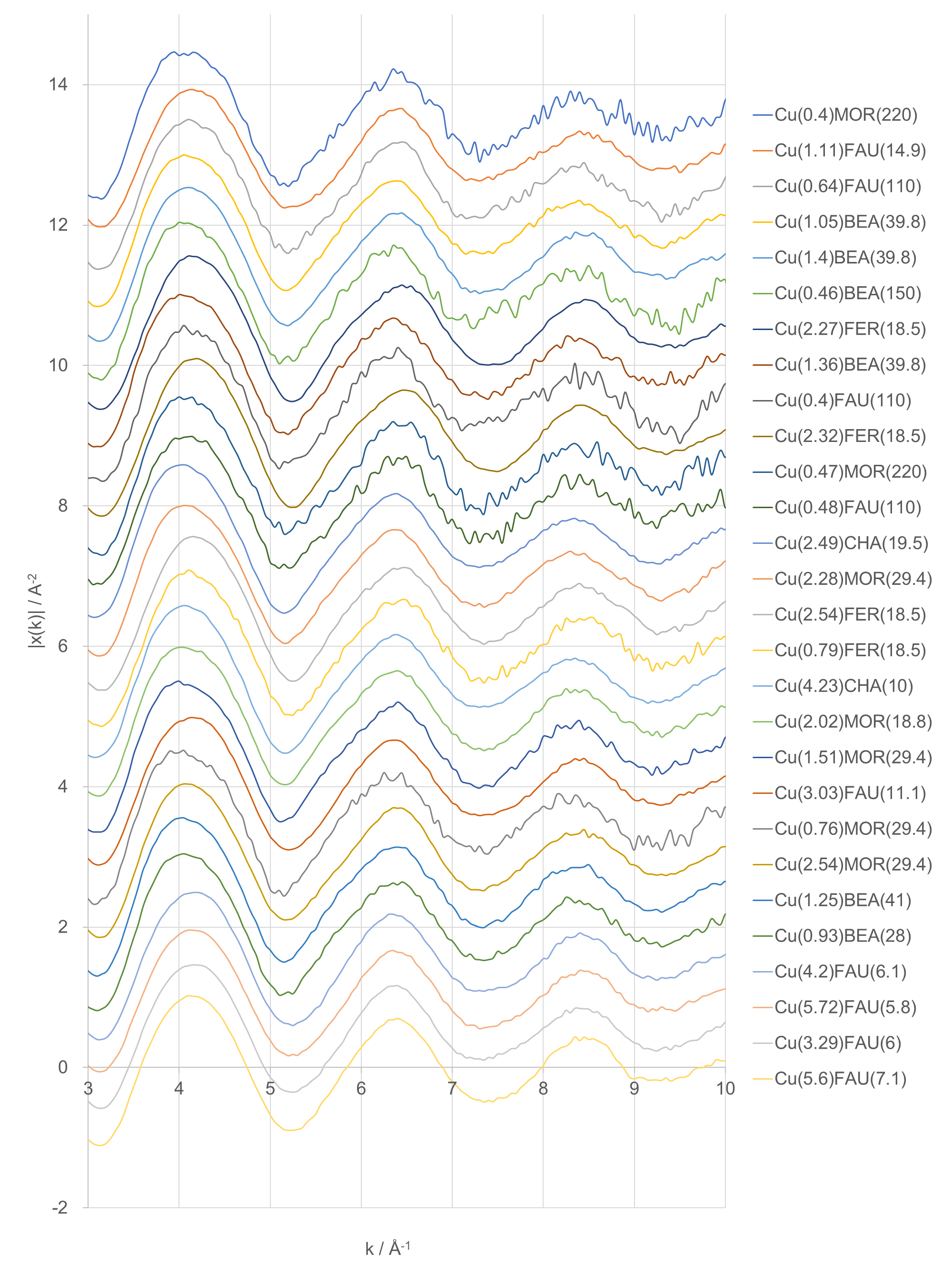


**Figure S9** Cu K edge EXAFS spectra for all Cu zeolites in Figure 1(d).

**Table S1.** Results of CH_4_-H_2_O_2_ reaction using Cu zeolites and H zeolites. *^a^*

| Catalyst *^b^* | Zeolite *^c^* | Framework type | Si/Al_2_ *^d^* (mol/mol) | Cu loading *^d^* (wt%) | MeOH *^e^*  (mmol L^-1^) | MeOOH *^e^*  (mmol L^-1^) | HCOOH *^e^*  (mmol L^-1^) | Total *^f^*  (mmol L^-1^) | Increment by Cu *^g^*  (mmol L^-1^) | Specific activity *^h^*  (mmol / (Cu-mol)^-1^) |
| --- | --- | --- | --- | --- | --- | --- | --- | --- | --- | --- |
| Cu(4.23)CHA(10) | CHA-10 | CHA | 10 | 4.2 | 0.12 | 0.32 | 0.00 | 0.44 | 0.42 | 199 |
| Cu(2.49)CHA(19.5) | CHA-20 | CHA | 19.5 | 2.5 | 0.04 | 0.32 | 0.00 | 0.36 | 0.35 | 282 |
| Cu(5.72)FAU(5.8) | HSZ-320HOA | FAU | 5.8 | 5.7 | 0.12 | 0.12 | 0.00 | 0.24 | 0.23 | 81 |
| Cu(3.29)FAU(6) | HSZ-330HUA | FAU | 6 | 3.3 | 0.03 | 0.11 | 0.00 | 0.14 | 0.12 | 73 |
| Cu(4.2)FAU(6.1) | HSZ-331HSA | FAU | 6.1 | 4.2 | 0.05 | 0.12 | 0.00 | 0.17 | 0.17 | 81 |
| Cu(5.6)FAU(7.1) | HSZ-341NHA | FAU | 7.1 | 5.6 | 0.03 | 0.11 | 0.00 | 0.14 | 0.14 | 50 |
| Cu(3.03)FAU(11.1) | HSZ-350HUA | FAU | 11.1 | 3.0 | 0.05 | 0.21 | 0.00 | 0.26 | 0.25 | 165 |
| Cu(1.11)FAU(14.9) | HSZ-360HUA | FAU | 14.9 | 1.1 | 0.04 | 0.71 | 0.00 | 0.75 | 0.74 | 1336 |
| Cu(0.64)FAU(110) | HSZ-385HUA | FAU | 110 | 0.6 | 0.02 | 0.40 | 0.00 | 0.42 | 0.37 | 1161 |
| Cu(0.4)FAU(110) | HSZ-385HUA | FAU | 110 | 0.4 | 0.01 | 0.12 | 0.00 | 0.13 | 0.08 | 399 |
| Cu(0.48)FAU(110) | HSZ-385HUA | FAU | 110 | 0.5 | 0.00 | 0.12 | 0.00 | 0.12 | 0.07 | 292 |
| Cu(2.02)MOR(18.8) | HSZ-640HOA | MOR | 18.8 | 2.0 | 0.27 | 0.57 | 0.00 | 0.83 | 0.20 | 196 |
| Cu(2.54)MOR(29.4) | HSZ-660HOA | MOR | 29.4 | 2.5 | 0.07 | 0.15 | 0.00 | 0.22 | 0.17 | 134 |
| Cu(2.28)MOR(29.4) | HSZ-660HOA | MOR | 29.4 | 2.3 | 0.12 | 0.23 | 0.00 | 0.35 | 0.30 | 264 |
| Cu(0.76)MOR(29.4) | HSZ-660HOA | MOR | 29.4 | 0.8 | 0.05 | 0.06 | 0.00 | 0.11 | 0.06 | 159 |
| Cu(1.51)MOR(29.4) | HSZ-660HOA | MOR | 29.4 | 1.5 | 0.08 | 0.10 | 0.00 | 0.18 | 0.13 | 173 |
| Cu(0.47)MOR(220) | HSZ-690HOA | MOR | 220 | 0.5 | 0.01 | 0.09 | 0.00 | 0.09 | 0.07 | 296 |
| Cu(0.4)MOR(220) | HSZ-690HOA | MOR | 220 | 0.4 | 0.02 | 0.31 | 0.00 | 0.33 | 0.31 | 1559 |
| Cu(2.54)FER(18.5) | HSZ-720NHA | FER | 18.5 | 2.5 | 0.19 | 0.23 | 0.00 | 0.42 | 0.33 | 256 |
| Cu(2.32)FER(18.5) | HSZ-720NHA | FER | 18.5 | 2.3 | 0.26 | 0.27 | 0.00 | 0.53 | 0.44 | 376 |
| Cu(2.27)FER(18.5) | HSZ-720NHA | FER | 18.5 | 2.3 | 0.33 | 0.27 | 0.00 | 0.59 | 0.50 | 441 |
| Cu(0.79)FER(18.5) | HSZ-720NHA | FER | 18.5 | 0.8 | 0.06 | 0.13 | 0.00 | 0.19 | 0.10 | 253 |
| Cu(0.93)BEA(28) | HSZ-930NHA | BEA | 28 | 0.9 | 0.01 | 0.06 | 0.00 | 0.07 | 0.04 | 86 |
| Cu(1.05)BEA(39.8) | HSZ-940HOA | BEA | 39.8 | 1.1 | 0.02 | 0.40 | 0.00 | 0.42 | 0.36 | 686 |
| Cu(1.36)BEA(39.8) | HSZ-940HOA | BEA | 39.8 | 1.4 | 0.01 | 0.34 | 0.00 | 0.35 | 0.29 | 426 |
| Cu(1.4)BEA(39.8) | HSZ-940HOA | BEA | 39.8 | 1.4 | 0.03 | 0.42 | 0.00 | 0.44 | 0.38 | 543 |
| Cu(1.25)BEA(41) | HSZ-940NHA | BEA | 41 | 1.2 | 0.01 | 0.06 | 0.00 | 0.07 | 0.06 | 96 |
| Cu(0.46)BEA(150) | JRC-Z-HB150 | BEA | 150 | 0.5 | 0.01 | 0.21 | 0.00 | 0.22 | 0.12 | 527 |
| Cu(3.32)MFI(23.9) | HSZ-822HOA | MFI | 23.9 | 3.3 | 0.57 | 0.29 | 0.00 | 0.86 | -5.71 | - |
| Cu(2.55)MFI(23.9) | HSZ-822HOA | MFI | 23.9 | 2.5 | 1.17 | 0.60 | 0.00 | 1.77 | -4.80 | - |
| Cu(1.66)MFI(37) | HSZ-840HOA | MFI | 37 | 1.7 | 0.91 | 0.39 | 0.00 | 1.30 | -4.63 | - |
| Cu(0.45)MFI(37) | HSZ-840HOA | MFI | 37 | 0.4 | 1.14 | 0.88 | 0.00 | 2.02 | -3.91 | - |
| Cu(1.47)MFI(37) | HSZ-840HOA | MFI | 37 | 1.5 | 1.24 | 0.78 | 0.00 | 2.02 | -3.91 | - |
| Cu(0.33)MFI(37) | HSZ-840HOA | MFI | 37 | 0.3 | 0.64 | 0.82 | 2.22 | 3.68 | -2.25 | - |
| Cu(1.85)MFI(39) | HSZ-840NHA | MFI | 37 | 1.9 | 1.23 | 0.60 | 0.00 | 1.83 | -6.90 | - |
| CHA(10) | Nikki, CHA-10 | CHA | 10 | - | 0.00 | 0.02 | 0.00 | 0.02 | - | - |
| CHA(19.5) | Nikki, CHA-20 | CHA | 19.5 | - | 0.00 | 0.01 | 0.00 | 0.01 | - | - |
| FAU(5.8) | HSZ-320HOA | FAU | 5.8 | - | 0.00 | 0.01 | 0.00 | 0.01 | - | - |
| FAU(6) | HSZ-330HUA | FAU | 6 | - | 0.01 | 0.01 | 0.00 | 0.02 | - | - |
| FAU(6.1) | HSZ-331HSA | FAU | 6.1 | - | 0.00 | 0.00 | 0.00 | 0.00 | - | - |
| FAU(7.1) | HSZ-341NHA | FAU | 7.1 | - | 0.00 | 0.00 | 0.00 | 0.00 | - | - |
| FAU(11.1) | HSZ-350HUA | FAU | 11.1 | - | 0.01 | 0.00 | 0.00 | 0.01 | - | - |
| FAU(14.9) | HSZ-360HUA | FAU | 14.9 | - | 0.00 | 0.01 | 0.00 | 0.01 | - | - |
| FAU(110) | HSZ-385HUA | FAU | 110 | - | 0.01 | 0.04 | 0.00 | 0.05 | - | - |
| MOR(18.8) | HSZ-640HOA | MOR | 18.8 | - | 0.10 | 0.53 | 0.00 | 0.63 | - | - |
| MOR(29.4) | HSZ-660HOA | MOR | 29.4 | - | 0.02 | 0.04 | 0.00 | 0.05 | - | - |
| MOR(220) | HSZ-690HOA | MOR | 220 | - | 0.01 | 0.01 | 0.00 | 0.02 | - | - |
| FER(18.5) | HSZ-720NHA | FER | 18.5 | - | 0.02 | 0.07 | 0.00 | 0.09 | - | - |
| BEA(28) | HSZ-930NHA | BEA | 28 | - | 0.00 | 0.03 | 0.00 | 0.03 | - | - |
| BEA(39.8) | HSZ-940HOA | BEA | 39.8 | - | 0.01 | 0.05 | 0.00 | 0.06 | - | - |
| BEA(41) | HSZ-940NHA | BEA | 41 | - | 0.00 | 0.01 | 0.00 | 0.01 | - | - |
| BEA(150) | JRC-Z-HB150 | BEA | 150 | - | 0.01 | 0.09 | 0.00 | 0.10 | - | - |
| MFI(23.9) | HSZ-822HOA | MFI | 23.9 | - | 0.54 | 0.83 | 5.20 | 6.57 | - | - |
| MFI(37) | HSZ-840HOA | MFI | 37 | - | 0.74 | 0.92 | 4.27 | 5.93 | - | - |
| MFI(39) | HSZ-840NHA | MFI | 39 | - | 0.68 | 0.84 | 7.21 | 8.73 | - | - |
| Blank *^h^* | - | - | - | - | 0.02 | 0.10 | 0.00 | 0.12 | - | - |

*^a^* Reaction conditions: CH_4_ 3.5 MPa, 30 wt % H_2_O_2_ 155 μl, H_2_O 3 ml, catalyst 10 mg, 60 °C, 1 h. *^b^* All catalysts are calcined at 700 °C under air for 1h before use for reactions. *^c^* CHA zeolites, JRC-Z-HB150, HSZ series are supplied from JGC Catalysts and Chemicals Ltd., The Catalysis Society of Japan, Tosoh Co., respectively. *^d^* Determined by ICP/XRF. *^e^* Determined by ^1^H-NMR analysis of reaction solutions after the reaction for 1 h. *^f^* Total product conc. of MeOH, MeOOH and HCOOH. *^g^* Difference in the total products conc. of Cu zeolites from the corresponding H zeolites. *^g^* Determined by dividing the increments by Cu loading. *^h^* The reaction result without catalyst.

**Table S2.** Fe loadings of several zeolites determined by ICP (catalogue values).

| Framework type | Zeolite | Fe wt% |
| --- | --- | --- |
| FAU | HSZ-387HUA (Similar product of 385HUA) | 0.007 |
| MOR | HSZ-640HOA | 0.014 |
| FER | HSZ-720NHA | 0.014 |
| MFI | HSZ-820NHA (ammonium type of HSZ-820HOA) | 0.014 |

**Table S3.** Results of CH_4_-H_2_O_2_ reaction using various metal-MFI zeolites. *^a^*

| Metal | Zeolite *^b^* | Framework type | MeOH *^c^*  (mmol L^-1^) | MeOOH *^c^*  (mmol L^-1^) | HCOOH *^c^*  (mmol L^-1^) |
| --- | --- | --- | --- | --- | --- |
| Mn | HSZ-840NHA | MFI | 0.90 | 0.84 | 7.88 |
| Fe | HSZ-840HOA | MFI | 0.63 | 0.52 | 33.06 |
| Co | HSZ-840NHA | MFI | 0.82 | 1.00 | 6.72 |
| Ni | HSZ-840HOA | MFI | 0.94 | 0.84 | 8.29 |
| Rh | HSZ-820NHA | MFI | 0.21 | 0.76 | 1.45 |
| Ag | HSZ-840NHA | MFI | 0.21 | 0.24 | 2.38 |

*^a^* Reaction conditions: CH_4_ 3.5 MPa, 30 wt % H_2_O_2_ 155 μl, H_2_O 3 ml, catalyst 10 mg, 60 °C, 1 h. *^b^* Supplied from Tosoh Co., respectively. *^c^* Determined by ^1^H-NMR analysis of reaction solutions after the reaction for 1 h.

**Table S4.** Structural parameters for CuO_4_, CuO_5_, or CuO_6_ model structures used for the FEFF calculation.

| Structure | Cu-O_a_ *^a^* | | Cu-O_b_ *^a^* | |
| --- | --- | --- | --- | --- |
|  | R_a_ *^b^* (Å) | CN_a_ *^c^* | R_b_ *^b^* (Å) | CN_b_ *^c^* |
| SP | 1.96 | 4 | - | - |
| dOh | 1.96 | 4 | 2.4 | 2 |
| dSPy | 1.96 | 4 | 2.4 | 1 |
| SPy, TBPy | 1.96 | 5 | - | - |

*^a^* Two Cu-O with different radial distance for the model structures. *^b^* Radial distance. *^c^* Coordination number.

**Table S5.** The values for the fifteen descriptors of the twenty-eight Cu zeolite catalysts together with the specific activity and classification by unsupervised machine learning.

| Catalyst | Explanatory variables | | | | | | | | | | | | | | | Specific activity  (mmol/(Cu-mol)^-1^) | Classified  specific activity *^a^* |
| --- | --- | --- | --- | --- | --- | --- | --- | --- | --- | --- | --- | --- | --- | --- | --- | --- | --- |
|  | FD | TD10 | DI | Da, Db, Dc | | | AV | CD | Si/Al2 | Cu wt | IE | SA | E at abs 0.5 | Int at 1.5 Å | Int at 2.1 Å |  |  |
| Cu(4.23)CHA(10) | 15.1 | 677 | 7.37 | 3.72 | 3.72 | 3.72 | 17.27 | 3 | 10 | 4.23 | 50 | 699 | 8987.0 | 1.270 | 0.323 | 199 | 2 |
| Cu(2.49)CHA(19.5) | 15.1 | 677 | 7.37 | 3.72 | 3.72 | 3.72 | 17.27 | 3 | 19.5 | 2.49 | 52 | 795 | 8987.4 | 1.268 | 0.331 | 282 | 2 |
| Cu(5.72)FAU(5.8) | 13.3 | 579 | 11.24 | 7.35 | 7.35 | 7.35 | 24.42 | 3 | 5.8 | 5.72 | 42 | 544 | 8986.5 | 1.213 | 0.268 | 81 | 2 |
| Cu(3.29)FAU(6) | 13.3 | 579 | 11.24 | 7.35 | 7.35 | 7.35 | 24.42 | 3 | 6 | 3.29 | 25 | 740 | 8986.5 | 1.221 | 0.254 | 73 | 2 |
| Cu(4.2)FAU(6.1) | 13.3 | 579 | 11.24 | 7.35 | 7.35 | 7.35 | 24.42 | 3 | 6.1 | 4.20 | 32 | 784 | 8986.5 | 1.274 | 0.283 | 81 | 2 |
| Cu(5.6)FAU(7.1) | 13.3 | 579 | 11.24 | 7.35 | 7.35 | 7.35 | 24.42 | 3 | 7.1 | 5.60 | 51 | 639 | 8986.8 | 1.305 | 0.308 | 50 | 2 |
| Cu(3.03)FAU(11.1) | 13.3 | 579 | 11.24 | 7.35 | 7.35 | 7.35 | 24.42 | 3 | 11.1 | 3.03 | 38 | 822 | 8986.8 | 1.253 | 0.285 | 165 | 2 |
| Cu(1.11)FAU(14.9) | 13.3 | 579 | 11.24 | 7.35 | 7.35 | 7.35 | 24.42 | 3 | 14.9 | 1.11 | 18 | 759 | 8986.1 | 1.146 | 0.224 | 1336 | 1 |
| Cu(0.64)FAU(110) | 13.3 | 579 | 11.24 | 7.35 | 7.35 | 7.35 | 24.42 | 3 | 110 | 0.64 | 68 | 839 | 8986.5 | 1.212 | 0.257 | 1161 | 1 |
| Cu(0.4)FAU(110) | 13.3 | 579 | 11.24 | 7.35 | 7.35 | 7.35 | 24.42 | 3 | 110 | 0.40 | 43 | 905 | 8987.4 | 1.323 | 0.320 | 399 | 0 |
| Cu(0.48)FAU(110) | 13.3 | 579 | 11.24 | 7.35 | 7.35 | 7.35 | 24.42 | 3 | 110 | 0.48 | 51 | 791 | 8986.7 | 1.223 | 0.297 | 292 | 0 |
| Cu(2.02)MOR(18.8) | 17 | 938 | 6.7 | 1.57 | 2.95 | 6.45 | 12.27 | 2 | 18.8 | 2.02 | 40 | 456 | 8987.1 | 1.265 | 0.324 | 196 | 2 |
| Cu(2.54)MOR(29.4) | 17 | 938 | 6.7 | 1.57 | 2.95 | 6.45 | 12.27 | 2 | 29.4 | 2.54 | 75 | 533 | 8986.8 | 1.290 | 0.310 | 134 | 2 |
| Cu(2.28)MOR(29.4) | 17 | 938 | 6.7 | 1.57 | 2.95 | 6.45 | 12.27 | 2 | 29.4 | 2.28 | 68 | 551 | 8987.2 | 1.263 | 0.298 | 264 | 2 |
| Cu(0.76)MOR(29.4) | 17 | 938 | 6.7 | 1.57 | 2.95 | 6.45 | 12.27 | 2 | 29.4 | 0.76 | 22 | 477 | 8987.1 | 1.280 | 0.306 | 159 | 2 |
| Cu(1.51)MOR(29.4) | 17 | 938 | 6.7 | 1.57 | 2.95 | 6.45 | 12.27 | 2 | 29.4 | 1.51 | 45 | 468 | 8987.1 | 1.277 | 0.326 | 173 | 2 |
| Cu(0.47)MOR(220) | 17 | 938 | 6.7 | 1.57 | 2.95 | 6.45 | 12.27 | 2 | 220 | 0.47 | 100 | 603 | 8987.4 | 1.267 | 0.351 | 296 | 0 |
| Cu(0.4)MOR(220) | 17 | 938 | 6.7 | 1.57 | 2.95 | 6.45 | 12.27 | 2 | 220 | 0.40 | 84 | 504 | 8987.1 | 1.219 | 0.292 | 1559 | 1 |
| Cu(2.54)FER(18.5) | 17.6 | 1021 | 6.31 | 1.56 | 3.4 | 4.69 | 10.01 | 2 | 18.5 | 2.54 | 49 | 377 | 8986.8 | 1.312 | 0.314 | 256 | 2 |
| Cu(2.32)FER(18.5) | 17.6 | 1021 | 6.31 | 1.56 | 3.4 | 4.69 | 10.01 | 2 | 18.5 | 2.32 | 45 | 383 | 8986.8 | 1.366 | 0.354 | 376 | 0 |
| Cu(2.27)FER(18.5) | 17.6 | 1021 | 6.31 | 1.56 | 3.4 | 4.69 | 10.01 | 2 | 18.5 | 2.27 | 45 | 330 | 8986.8 | 1.346 | 0.337 | 441 | 0 |
| Cu(0.79)FER(18.5) | 17.6 | 1021 | 6.31 | 1.56 | 3.4 | 4.69 | 10.01 | 2 | 18.5 | 0.79 | 16 | 243 | 8986.7 | 1.328 | 0.323 | 253 | 2 |
| Cu(0.93)BEA(28) | 15.3 | 805 | 6.68 | 5.95 | 5.95 | 5.95 | 20.52 | 3 | 28 | 0.93 | 27 | 576 | 8987.2 | 1.296 | 0.350 | 86 | 2 |
| Cu(1.05)BEA(39.8) | 15.3 | 805 | 6.68 | 5.95 | 5.95 | 5.95 | 20.52 | 3 | 39.8 | 1.05 | 42 | 635 | 8987.2 | 1.245 | 0.294 | 686 | 0 |
| Cu(1.36)BEA(39.8) | 15.3 | 805 | 6.68 | 5.95 | 5.95 | 5.95 | 20.52 | 3 | 39.8 | 1.36 | 54 | 534 | 8987.1 | 1.283 | 0.314 | 426 | 0 |
| Cu(1.4)BEA(39.8) | 15.3 | 805 | 6.68 | 5.95 | 5.95 | 5.95 | 20.52 | 3 | 39.8 | 1.40 | 55 | 589 | 8986.8 | 1.292 | 0.330 | 543 | 0 |
| Cu(1.25)BEA(41) | 15.3 | 805 | 6.68 | 5.95 | 5.95 | 5.95 | 20.52 | 3 | 41 | 1.25 | 51 | 577 | 8987.2 | 1.302 | 0.363 | 96 | 2 |
| Cu(0.46)BEA(150) | 15.3 | 805 | 6.68 | 5.95 | 5.95 | 5.95 | 20.52 | 3 | 150 | 0.46 | 66 | 577 | 8987.1 | 1.316 | 0.312 | 527 | 0 |

*^a^* The specific activity in low, medium and high groups is indicated by the numbers of 2, 0, and 1, respectively.
